# mTOR hypoactivity leads to trophectoderm cell failure by enhancing lysosomal activation and disrupting the cytoskeleton in preimplantation embryo

**DOI:** 10.1186/s13578-023-01176-3

**Published:** 2023-11-30

**Authors:** Chiyuan Ma, Qin Li, Yuxin Yang, Lei Ge, Jiaxuan Cai, Juan Wang, Maoxian Zhu, Yue Xiong, Wenya Zhang, Jingtong Xie, Yujing Cao, Huashan Zhao, Qing Wei, Chen Huang, Junchao Shi, Jian V. Zhang, Enkui Duan, Xiaohua Lei

**Affiliations:** 1 Center for Energy Metabolism and Reproduction, Shenzhen Institute of Advanced Technology, Chinese Academy of Sciences, Shenzhen, 518055 China; 2 School of Basic Medical Sciences and Life Sciences, Hainan Medical University, Haikou, 571199 China; 3 State Key Laboratory of Stem Cell and Reproductive Biology, Institute of Zoology, Chinese Academy of Sciences, Beijing, 100101 China; 4 CAS Key Laboratory of Genome Sciences and Information, China National Center for Bioinformation, Beijing Institute of Genomics, Chinese Academy of Sciences, Beijing, 100101 China

**Keywords:** mTOR, Trophectoderm cell failure, Transcriptome, DNA methylation, Lysosomal activation, Actin cytoskeleton disruption

## Abstract

**Background:**

Metabolic homeostasis is closely related to early impairment of cell fate determination and embryo development. The protein kinase mechanistic target of rapamycin (mTOR) is a key regulator of cellular metabolism in the body. Inhibition of mTOR signaling in early embryo causes postimplantation development failure, yet the mechanisms are still poorly understood.

**Methods:**

Pregnancy mice and preimplantation mouse embryo were treated with mTOR inhibitor in vivo and in vitro respectively, and subsequently examined the blastocyst formation, implantation, and post-implantation development. We used immunofluorescence staining, RNA-Seq smart2, and genome-wide bisulfite sequencing technologies to investigate the impact of mTOR inhibitors on the quality, cell fate determination, and molecular alterations in developing embryos.

**Results:**

We showed mTOR suppression during preimplantation decreases the rate of blastocyst formation and the competency of implantation, impairs the post implantation embryonic development. We discovered that blocking mTOR signaling negatively affected the transformation of 8-cell embryos into blastocysts and caused various deficiencies in blastocyst quality. These included problems with compromised trophectoderm cell differentiation, as well as disruptions in cell fate specification. mTOR suppression significantly affected the transcription and DNA methylation of embryos. Treatment with mTOR inhibitors increase lysosomal activation and disrupts the organization and dynamics of the actin cytoskeleton in blastocysts.

**Conclusions:**

These results demonstrate that mTOR plays a crucial role in 8-cell to blastocyst transition and safeguards embryo quality during early embryo development.

**Supplementary Information:**

The online version contains supplementary material available at 10.1186/s13578-023-01176-3.

## Introduction

Early pregnancy loss is a common but not easily detected event for gestational women. It is believed that, in many cases, early miscarriages or abnormal pregnancies are caused by dysregulation of preimplantation development [1]. During early pregnancy, disorders of nutritional metabolism can induce cellular and molecular alterations in embryos, thereby affecting embryonic development, maternal–fetal interactions, and postnatal health [2, 3]. Despite the nutritional and metabolic requirement of preimplantation development have been shown to be minimal, some primary nutrients (for example, pyruvate, lactate and glucose) or microenvironment cues (oxygen tension) are essential for the zygotic transition and for the morula stage transition, which are two key early preimplantation events in initiating zygotic genome activation (ZGA) and cell fate commitment [4–6]. Undernutrition causes molecular and metabolic adaptations of embryos to cope with nutritional deficiencies or excesses for blastocyst formation, while it may compromise the quality of the embryos, resulting in early pregnancy loss [7, 8]. Thus, well-balanced maternal nutrient metabolism is critical for embryo development.

The mechanistic target of rapamycin (mTOR) is an evolutionary conserved serine-threonine kinase that integrates a diversity of intracellular and extracellular signals, and mTOR regulate both proliferation and cell size in the early embryo and embryonic stem cells [9, 10]. In mammals, mTOR constitutes the catalytic subunit of two different multi-molecular complexes, namely rapamycin-sensitive mTOR complex 1 (mTORC1) and rapamycin-insensitive mTOR complex2 (mTORC2) [11]. mTORC1 regulates the balance between catabolism and anabolism in the cells by integrating information about the nutritional abundance and environmental status, while mTORC2 controls the actin cytoskeleton behavior and activates several pro-survival pathways [12]. In female reproduction, the mTOR regulates many cellular processes, including folliculogenesis [13], oocyte meiotic maturation [14], ovarian somatic cell proliferation [15] or the embryo’s response to intracellular and extracellular nutrition [16, 17]. While the viability and recruitment of primordial oocytes into the growing oocyte cohort do not depend on mTOR-dependent pathways, inhibiting mTOR signaling during the primordial oocyte stage can negatively affect oocyte genomic integrity, ovarian follicular development, and oocyte quality [14]. Recently, it has been reported that mTOR signaling controls lifespan and influence aging-related processes [18], such as stem cell function and embryonic diapause [19]. Treatment with an mTOR inhibitor reversibly halts mouse blastocyst development and preserves the pluripotent state of the remaining cells [20].

Notably, mTOR-deficient embryos in mouse lead to early postimplantation development failure, which indicates an indispensable role of mTOR signaling during early mammalian embryogenesis [21, 22]. Despite long-term inactivation of mTOR in results in embryonic lethality, it remains understood whether and how inhibition of mTOR signaling in the preimplantation stage affects pre- and peri-implantation embryonic development. In the present study, we employ a pharmacological approach both in vitro and in vivo to investigate the consequences of mTOR hypoactivity for early embryonic development in mouse. We show that mTOR inhibition in preimplantation significantly reduced the ratio of embryos developed to the blastocysts. Inhibiting mTOR prevents the blastocyst transformation from the 8-cell/morula stage and compromises the blastocyst quality. We also find that mTOR suppression affect cell fate determination and disrupt the trophectoderm cell proliferation and differentiation by inducing transcriptome and epigenetic alterations of embryo. Specifically, treatment with mTOR inhibitor increase lysosomal activation and disrupts the organization and dynamics of the actin cytoskeleton in embryos.

## Methods

### Animals and feeding treatment

ICR male (10 week old) and female mice (8–10 week old) were obtained from Beijing Vital River Laboratory Animal Technology Co., Ltd. Mice were housed in the Shenzhen Institutes of Advanced Technology, Chinese Academy of Sciences, according to the guidelines of the Committee for laboratory animals (No: SIAT-IACUC-210115-YYS-LXH-A1514). All mice used in this study were lived under an environment of 12 h light/dark cycle and 20–25 °C. Females were injected with 5 IU pregnant mare serum gonadotrophin (PMSG) to stimulate follicular development, followed by 5 IU human chorionic gonadotrophin (hCG) 46–48 h after PMSG and then mating with fertile male. The presence of a vaginal plug was used to determine pregnancy, with the day of the plug classified as 0.5 days post coitum (dpc).

### Embryo collection

Preimplantation embryos in 1-cell (at 24 h post-hCG), 2-cell (at 48 h post-hCG), 4-cell (at 54–56 h post-hCG), 8-cell (at 68–70 h post-hCG), morula (at 76–78 h post-hCG) and blastocyst stage (92–100 h post-hCG) were respectively flushed from oviducts or uterus with M2 medium (Millipore), embryos were washed and prepared for detection, drug treatment or culture.

### Drug treatment and embryo culture

For drug administration in vivo, the pregnancy mice at 0.5 dpc were administered with Rapalink-1 (APExBIO) through intraperitoneal injection at a dose of 1.5 mg/kg per day for two days. For drug treatment in vitro, washed 1-cell or 8-cell embryos were transferred to 96-well plate (Corning) respectively, which contains 100 μl KSOM (MR-107-D, Millipore) supplement with concentrations of 50, 100, 200 nM Rapalink-1 (RPL, APExBIO), 200 nM Rapamycin (RPY, LC Laboratories), and 5 μM JR-AB2-011(JR, MCE) for culturing in an incubator with 5% CO_2_ at 37 °C (1-cell embryos culture for 72 or 96 h) or (8-cell embryos culture for 24 or 48 h). Embryos cultured in KOSM medium without drug treatment were control (Con) group. The bright field image of embryos each group were captured every 24 h to monitor the developmental characteristics by a Nikon inverted Eclipse TS100 microscope equipped with a Digital Sight camera system (Nikon). At the end of the embryo culture, we counted the number of embryos at each developmental stage and the blastocyst formation rate was measure in each group.

### Immunofluorescence staining and confocal microscopy

The mouse embryos staining was performed as our previously described with minor modifications [23]. In brief, embryos were fixed with 3.7% paraformaldehyde (PFA) for 20 min or the embryos were removed the zona pellucida by incubation with Acidic Tyrode’s Solution (Sigma-Aldrich) before 3.7% PFA fixation. Following fixation, embryos were permeated with 0.25% Triton X-100 for 20 min at room temperature. After blocking 1 h at room temperature in 5% donkey serum diluted in PBS containing 0.05% tween-20, embryos were stained overnight at 4 °C with primary antibody for p-mTOR (1:200 dilution; CST), p-AKT (1:200 dilution; CST), p-4EBP1(1:200 dilution; CST), CDX2 (1:200 dilution; Biogenex), SOX2 (1:200 dilution; Abcam), NANOG (1:200 dilution; Abcam), SOX17 (1:200 dilution; R&D), E-cadherin (1:200 dilution; R&D), Lamp1, Lamp2, LC3 and fluorescein isothiocyanate labeled phalloidin (FITC-phalloidin, Sigma). After three time washed with PBS containing 0.05% tween-20 and 0.5% donkey serum, the embryos were incubated with Alexa Fluor 488-conjugated donkey anti-mouse (1:200 dilution, Thermo Fisher Scientific), Alexa Fluor 568-conjugated donkey anti-rabbit (1:200 dilution, Thermo Fisher Scientific) or Alexa Fluor 647-conjugated donkey anti-goat (1:200 dilution, Thermo Fisher Scientific) secondary antibodies and Hoechst33342 (1:500, Sigma) for 1 h at room temperature. After washed three times, embryos were mounted on slides with a drop of antifading agent (DABCO™, Sigma) and immunofluorescence was captured by laser-scanning confocal microscope (LSM880, Zeiss). Images were always acquired using the same confocal microscope settings in the same molecules.

### LysoTracker assay

The embryos were incubated in M2 containing 1 μM LysoTracker Green (Invitrogen) for 1 h. After that, they were rinsed with fresh M2 multiple times before being imaged using confocal microscopy.

### TUNEL staining for blastocyst

To detect the cellular apoptosis in the blastocyst, 3.7% PFA fixed blastocysts were collected and TUNEL staining was performed using the TMR red in situ cell death detection kit (Roche) as described in instruction manual. DNA was counterstained with Hoechst33342 nuclear stain (Sigma). Fluorescence images were captured using a LSM880 (Zeiss) or Leica TCS SP8 (Leica) confocal microscope, and the red positively stained cell within the blastocysts were counted.

### 3D fluorescence images of blastocyst and cell counting

Fluorescence images of blastocyst were acquired using a Zeiss LSM880 or Leica TCS SP8 and optical sections were obtained every 2 μm. Briefly, as our previously described [23], 40× water objectives and 488 and 543 nm laser light was applied. For three-dimensional (3D) reconstructions, IMARIS software (Bitplane) was then used to outline cell membranes and then create 3D models of all cells within the embryo. Cells were then scored according to their relative position, cells completely surrounded by others were denoted as inner cells while those that had an outer surface were denoted as outer cells. The cell number were counted with Imaris (Version 9.0.2) software.

### Embryo transfer and examination of implantation sites

The quality of blastocysts developed from mTOR inhibitor treatment was further examined via embryo transfer. After cultivation, some blastocysts of mTOR inhibitor treatment group and control group were collected and were transferred to the uteri of pseudopregnant recipient mice at 2.5 dpc. Ten to fifteen embryos were randomly transferred per recipient. The number of implantation sites (IS) on day 5.5 or 7.5 dpc were counted by intravenous injection of 0.1 ml 1% Chicago Sky Blue solution (Sigma). The size of IS were examined and the fetuses at different developmental stage were collected for evaluation.

### Library preparation, RNA sequencing and analysis

#### Library preparation, construction, and sequencing

The blastocysts were transferred to PCR tubes with the RNA lysis reagents (one blastocyst/tube) and were lysed to release all RNAs, which were then reverse transcribed into first strand cDNAs by using the Smart‐Seq2 method. Single embryo RNA‐Seq was performed in Annoroad Gene Technology Co. Ltd (Beijing) according to previously descript method [24]. Briefly, the first strand cDNA was synthesized with reverse transcription, and the second-strand cDNA was synthesized via PCR amplification. These cDNAs were pre‐amplified and then purified to construct the cDNA library. Key steps include cDNA fragmentation and end‐repair (i.e., a single A base was added to the 3ʹend). After library construction, the insert size was assessed using the Agilent Bioanalyzer 2100 system (Agilent Technologies, CA, USA), and the qualified insert size was accurately quantified by using the StepOnePlus Real-Time PCR System (Library valid concentration > 2 nM). The constructed libraries were sequenced on an Illumina HiSeq 2500 platform, while 125 bp paired‐end reads were generated.

#### Filter and alignment of sequencing reads, IGV and FPKM

The raw reads were filtered with a quality control pipeline in Perl script to remove the Trim Smart-seq2 public primer sequence from the reads. Reads were discarded if the length of trimmed reads was less than 30 bp. With PE sequence, both reads were removed, and the low-quality reads (i.e., the number of reads bases whose Phred quality value was less than or equal to 19 accounted for more than 15%). If either one of the PE reads was defined as low quality, we will remove both ends of the reads). The contaminated reads for adapters were trimmed off. We also removed the reads whose N base was more than 5% for total bases. If either of the PE reads had high N bases, both ends of the reads were removed. We mapped the filtered reads to the mouse reference genome using HISAT2 (version 2.1.0) [25]. Integrative Genomics Viewer (IGV) was used to visualize the results of mapping with a heatmap, histogram, scatter plot or other style. The read count for each gene in each sample was counted with HTSeq v0.6.0, and the Fragments per kilobase millon mapped reads (FPKM) value was then calculated to estimate the expression level of genes in each sample.

#### Analysis of differential gene expression

Differentially expressed genes (DEGs) were identified using DESeq2 v1.6.3. DESeq2, to calculate the expression level of each gene per sample by using the linear regression, and the p-value was calculated with the Wald test. The *p*-value was corrected by the BH method. Genes with |log2(fold change)|≥ 1 and adjust *p*-value ≤ 0.05 are identified as differentially expressed genes (DEGs).

#### Functional annotation and enrichment analysis

Ensembl Gene IDs from each group were uploaded to the DAVID Functional Annotation Tool (http://david.abcc.ncifcrf.gov/; Version 6.7). All significant DEGs between the transcriptomes of embryos subjected to RPL treated and those without treated (Control) were subjected to gene ontology (GO) and Kyoto Encyclopedia of Genes and Genomes (KEGG) pathway analysis. Briefly, the GO (Gene Ontology, http://geneontology.org/) and KEGG (http://www.kegg.jp/) enrichment of DEGs was implemented by the use of hypergeometric test, in which *p*-value is calculated and adjusted as q-value, and the background data are genes in the whole genome. GO terms with *q* < 0.05 were significantly enriched and specifically enriched pathways are shown. GSEA software was used to conduct enrichment analysis.

### Genome-wide bisulfite sequencing (BS-seq) and analysis

BS-seq was performed as our previously described [23]. Briefly, before BS-seq, lysis buffer and 0.5 μl proteinase K were added into the PCR tubes and the blastocyst derived from different groups was placed into the tube (two embryo/tube). Bisulfite conversion was performed on cell lysates using EZ DNA Methylation-Gold Kit (Zymo Research); the converted DNA was then purified, library construction and library quantification. Qualified libraries were prepared for 125-bp paired-end sequencing on HiSeq2500 platform. The bisulfite conversion, libraries construction and amplification and sequencing procedures were performed by Annoroad Gene Technology Co., Ltd., Beijing, China.

For DNA methylation level estimation, after deduplication, the DNA methylation level was determined by the ratio of the number of reads supporting C (methylated) to that of total reads (methylated and unmethylated), which produced by Bismark toolkit of bismark methylation extractor. In the DNA methylome analysis, CpG sites with greater than 90% DNA methylation were considered methylated, whereas CpG sites with less than 10% DNA methylation levels were considered unmethylated. The read coverage threshold used to call the DNA methylation level for any cytosine was 3× for the samples. Differentially methylated regions between two groups were identified by DSS R packages (version 2.14.0 l) with default parameters. The annotation for the DMRs were conducted by BED tools (version 2.20.1). The GO and KEGG enrichment analysis was performed with the online tool (http://david.abcc.ncifcrf.gov/). IPA software was used for the canonical signaling pathway analysis. Functional gene sets were downloaded from the web http://software.broadinstitute.org/gsea/index.jsp), and GSEA software was used to conduct enrichment analysis.

### Statistical analysis

All experiments were repeated at least three times. The statistical analyses were performed using GraphPad Prism 5 (GraphPad Software, La Jolla, CA). Unless stated otherwise, the results are showed as the mean ± s.e.m. One-way ANOVA or Student’s t test was used to determine the level of significance. *p* < 0.05 is considered statistically significant, and *p* < 0.01 is considered statistically very significant.

## Results

### Inhibition of mTOR signaling during preimplantation impairs the development of embryos in vitro and in vivo

**Fig. 1:**
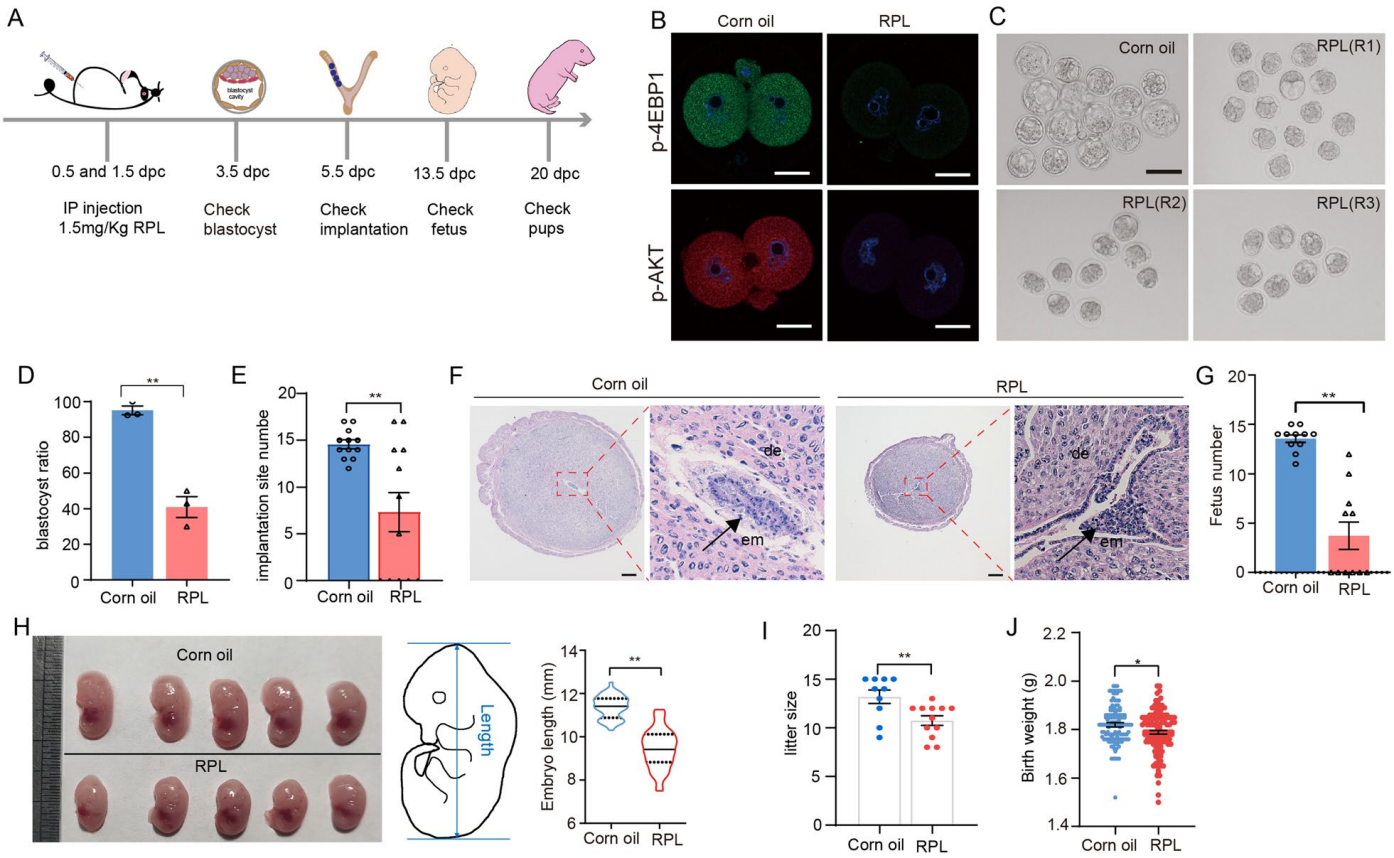
Developmental phenotypes of embryos from pregnancy mouse that were inhibition of mTOR signaling during preimplantation development. **A** Schematic illustration of the experimental setup of mouse treatment, embryo recovery and detection. **B** Immunostaining showing the levels of pS6 and p-AKT after 24 h of treatment with mTOR inhibitors at the 1-cell stage. Scale bars, 20 μm. **C** Representative bright-field images of embryos recovered from corn oil and RPL treated mice at 3.5 dpc. Scale bars, 100 μm. **D** The percentage of blastocysts was calculated at 3.5 dpc (n = 3). **E** Assessment of the implantation potential of blastocysts derived from corn oil and RPL treated mice (n = 12). **F** HE stained sections of paraffin-embedded embryos at 5.5 dpc. em, embryo. de, decidua. **G** Calculation of the number of fetuses recovered from the uteri at 13.5 dpc (n = 11). **H** Representative images showing conceptuses at 13.5 dpc and quantification of embryo crown-rump length (n = 43 for Corn oil and 64 for RPL). Production of offspring (**I**) (n = 10 for Corn oil and 12 for RPL) and the body weights (**J**) of offspring (n = 127 for Corn oil and 131 for RPL) from corn oil and RPL treated embryos. Error bars are mean ± SEM. ** *p* < 0.01, * *p* < 0.05, n.s., not significant (*p* > 0.05). *RPL* Rapalink-1, *dpc* days postcoitus, *HE* Hematoxylin and eosin

To examine the dynamic changes and the role of mTOR signaling during preimplantation development, we first performed immunofluorescent staining for phosphor-mTOR, phospho-4E-BP1 Thr37/46 (P-4E-BP1) and phospho-Akt Ser473 (P-AKT) in vivo developed mouse embryos. The results showed that mTOR signaling is present throughout all stages of preimplantation embryo development, with a noticeable decrease observed at the blastocyst stage (Additional file 1: Figure S1). We investigated whether inhibition of mTOR activity in preimplantation would affect embryonic development. For this purpose, 1.5 mg/Kg of RPL (Rapalink-1, the third-generation bivalent mTOR inhibitor [26, 27]) was injected into pregnancy mice at 0.5 and 1.5 dpc with by abdominal injection respectively, and subsequently examined the blastocyst formation, implantation, and post-implantation development (Fig. 1A). As expected, mTOR inhibitor treatment in vivo significantly reduced both the level of P-4E-BP1 (a marker of mTORC1 signaling) and P-AKT (a marker of mTORC2 signaling) (Fig. 1B). Additionally, a noticeable decrease in the rate of blastocyst formation was observed when compare with untreated group (41% for RPL vs 95% for Corn oil), although a majority of embryos were able to reach the morula stage (Fig. 1C and D). We next detected embryo implantation at 5.5 dpc (day-postcoitum). The IS number was significantly lower in the group treated with mTOR inhibitor (~ 7) than that in the corn oil control group (~ 14) (Fig. 1E) and appeared morphologically abnormal (Fig. 1F). In accordance with the reduced implantation sites number, fetus number were also decreased at 13.5 dpc (Fig. 1G), as measured by the crown-rump length of surviving embryos (Fig. 1H). Additionally, concordant with the developmental defects of the embryos, mTOR treatment mice had a lower litter size (Fig. 1I) and birth weight than untreated mice (Fig. 1J).

**Fig. 2:**
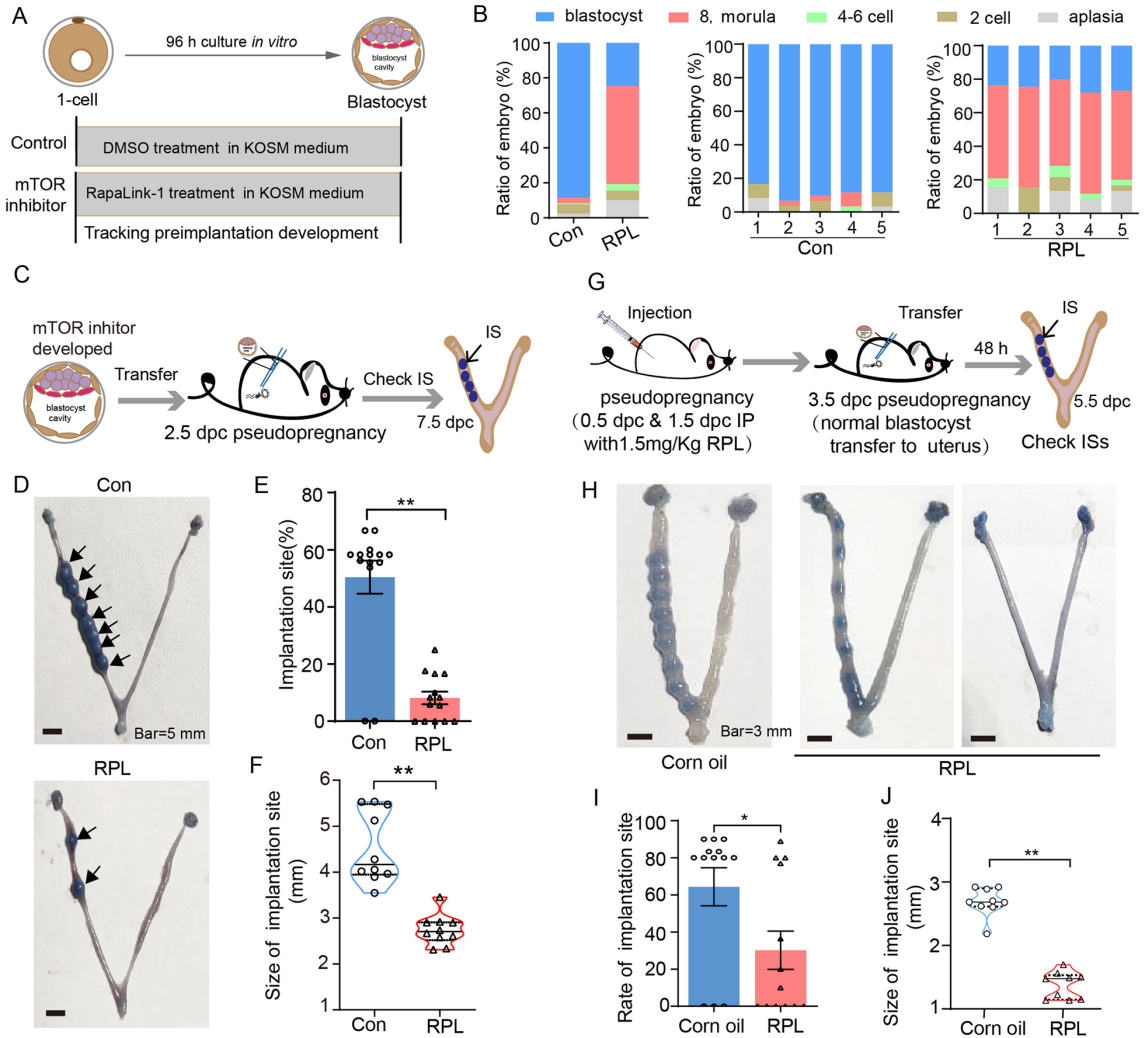
Detrimental effect of preimplantation mTOR suppression in vitro on mouse embryonic development. **A** Schematic of experimental setup whereby 1-cell embryos were cultured in KSOM with mTOR inhibitor treatment and without treated. **B** The rate of aplasia, 2-cell, 4–6 cell, 8-cell-morula, and blastocyst after 96 h cultured in Rapalink-1 treated group (RPL) and control group (Con). **C** Workflow of embryo transfer derivation from untreated or mTOR-inhibitor-developed blastocyst for identifying development potential. **D** Representative images of the implantation sites between Con and RPL at 7.5 dpc. Scale bars, 5 mm. The rate of implantation sites (**E**) (n = 14) and the size of the implantation site (**F**) (n = 10) were quantified. **G** Overview of the normal blastocysts was transferred into pseudopregnant mice which were treated with RPL or corn oil for identifying the potential of uterine receptivity and embryo implantation. **H** Representative images of the implantation sites after 48 h transferred in pregnant mice treated with Rapalink-1 or corn oil. Scale bars, 3 mm. The number (**I**) (n = 13) and size (**J**) of implantation sites (n = 9) were calculated in corn oil and RPL group. Error bars are mean ± SEM. ^**^ *p* < 0.01, ^*^ *p* < 0.01, n.s., not significant (*p* > 0.05)

We further investigate whether suppression of mTOR signaling in vitro also affects blastocyst formation and embryonic development, 1-cell embryos were collected and cultured with 200 nM RPL for 96 h in KOSM medium (Fig. 2A). We found that most embryos can develop to the 8-cell/morula stage in RPL group, but the ratio of the blastocyst stage was significantly lower than that of the control group (Con), and with a concentration-dependent manner (Fig. 2B and Additional file 1: Figure S2A, B). To evaluate the blastocyst viability and developmental potential, we transfer the embryos into surrogate females (10 embryos to into one recipient female) to check embryo implantation and ISs (Fig. 2C). We found that the implantation rate of blastocyst developed from mTOR suppression was significantly lower that of the control group (50% vs 10%) (Fig. 2D and E), with a remarkable decreased in the size of the ISs (Fig. 2F).

The above results led us to ask whether the mTOR signaling suppression in mice adversely influenced uterine receptivity, we injected Rapalink-1 intraperitoneally into the pseudopregnant mice, then transfer of the normal blastocyst into the uterus and followed by checking implantation status 48 h later (Fig. 2G). The result showed that intraperitoneal administration of mTOR inhibitor impaired embryo implantation rates by showing increased number of mice without ISs (Fig. 2H and I). The size of ISs was significantly reduced in mTOR inhibitor treatment (Fig. 2J).

Collectively, these data indicate that suppression of mTOR signaling in preimplantation can decrease the rate of blastocyst formation, and impair the competency of implantation and fetal development.

### mTOR signal inhibition influence cell fate commitment of embryonic differentiation and compromise blastocysts quality

**Fig. 3:**
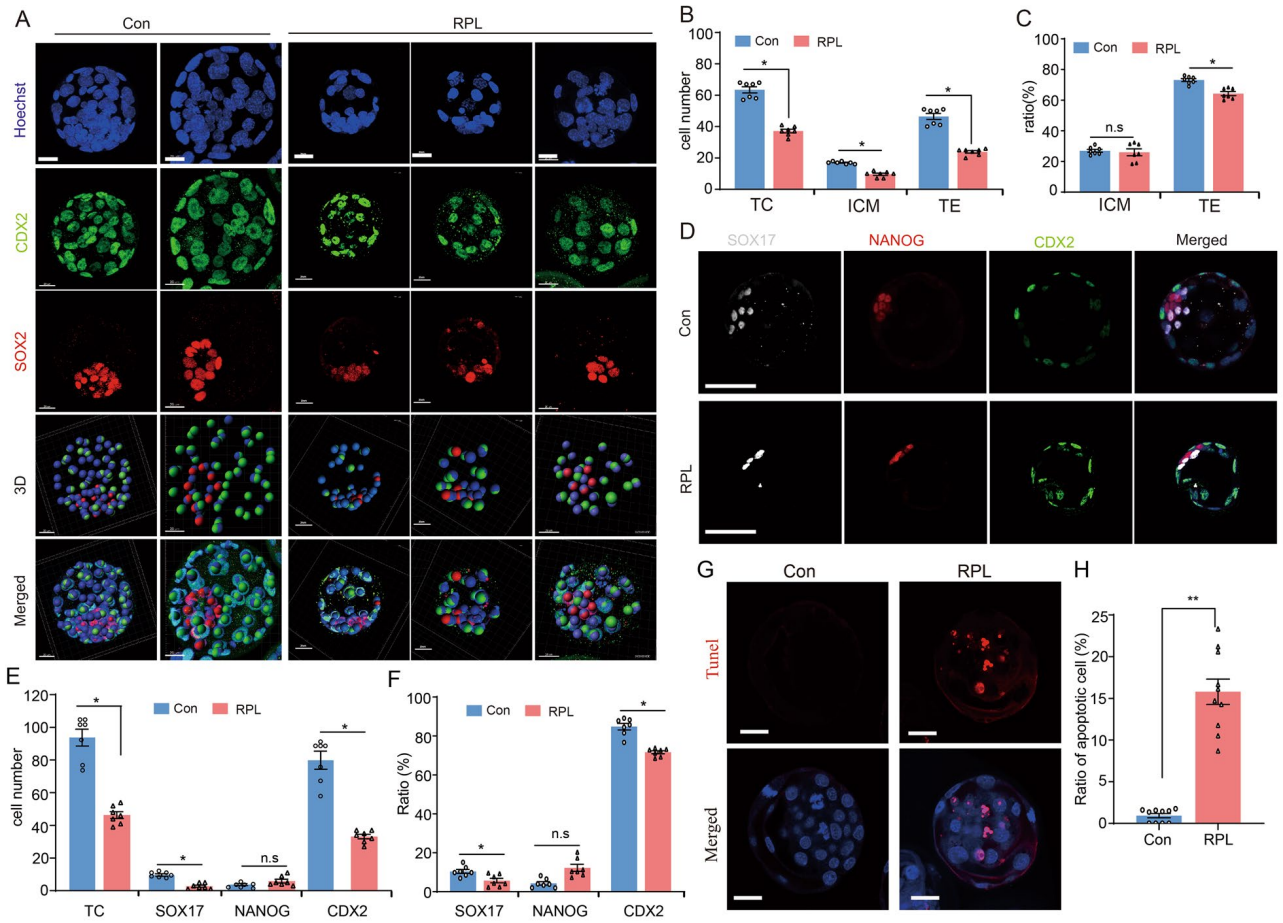
Assessment of the cell fate specification in blastocyst stage and the quality of blastocysts derive from mTOR inhibition embryos. **A** Representative 3D images of CDX2 and SOX2 immunofluorescence of blastocysts developed from the mTOR inhibitor treatment and without treated conditions. SOX2 positive cells represent the ICM (red), while CDX2 positive cells represent the TE (green). Nuclei were stained with Hoechst33342 (blue). Scale bars, 20 μm. **B** The number of TC, ICM, and TE of blastocysts in the RPL and Con group (n = 7). **C** The percentage of ICM cells and TE cells in the RPL and Control group (n = 7). **D** Representative immunofluorescence images of SOX17, NANOG and CDX2 of blastocyst derivation from the RPL treatment and Con group. Scale bars, 50 μm. **E** The cell number of total, SOX17^+^, NANOG^+^ and CDX2^+^ of blastocysts in the RPL and Con group (n = 7). **F** The percentage of SOX17^+^, NANOG^+^ and CDX2^+^ cells in each group (n = 7). **G** Terminal deoxynucleotidyl transferase-mediated dUTP nick end labeling (TUNEL) staining of the blastocysts derived from the RPL treatment and Control group. Scale bars, 50 μm. **H** The graph showing the quantification of the apoptosis cells (n = 10). *RPL* Rapalink-1, *Con* Control. *TC* total cell number, *ICM* inner cell mass, *TE* trophectoderm. Error bars are mean ± SEM. ^*^p < 0.05, ^**^ *p* < 0.01, n.s., not significant (*p* > 0.05)

Considering mTOR inhibition influences the cell proliferation and cell fate commitment, we carried out immunostaining of SOX2 (ICM marker) and CDX2 (TE marker). We observed a normal localization pattern of the SOX2^+^ and the CDX2^+^ cells in blastocyst using a 3D imaging system that SOX2 was specifically expressed in the ICM progenitors, but mTOR suppression reduced the size and diameter of the blastocyst (Fig. 3A). Additionally, we found that mTOR treatment significantly decreased the total cell numbers, ICM cell numbers and TE cell numbers in blastocyst (Fig. 3B). Importantly, there was a significantly decrease in the percentage of CDX2^+^ cells in mTOR treated embryos, but the percentage of SOX2^+^ cells was no significantly difference in mTOR treated embryo compare with control (Fig. 3C). These results indicate that polarization of ICM and TE was not disrupted, while mTOR inhibition has a detrimental effect on the quality of blastocyst and TE development.

To further confirm the notion, we examined the second differentiation event. The epiblast (EPI) progenitors expressing NANOG and primitive endoderm (PrE) progenitors expressing SOX17 can be observed in the ICM of blastocyst both in RPL treated and Con group (Fig. 3D). Remarkably, inhibition of mTOR activity enables a reduction in the total cell number, SOX17^+^ cell number and CDX2^+^ cell number (Fig. 3E). In addition to cell number, we observed a significant decrease in SOX17^+^ cell and CDX2^+^ cell proportion in RPL treated embryos compared with control embryos (Fig. 3F). TUNEL staining showed a significantly increase of apoptosis in RPL treated blastocyst, which was five times of the TUNEL-positive cells compare with control (Fig. 3G and H). Overall, these results suggest that mTOR suppression influences cell number, cell fate commitment of differentiation and compromised the quality of blastocyst.

### Inhibition of mTOR signaling by Rapalink-1 impairs the 8-cell/morula to blastocyst transformation and disrupts the balance of ICM/TE commitment

**Fig. 4:**
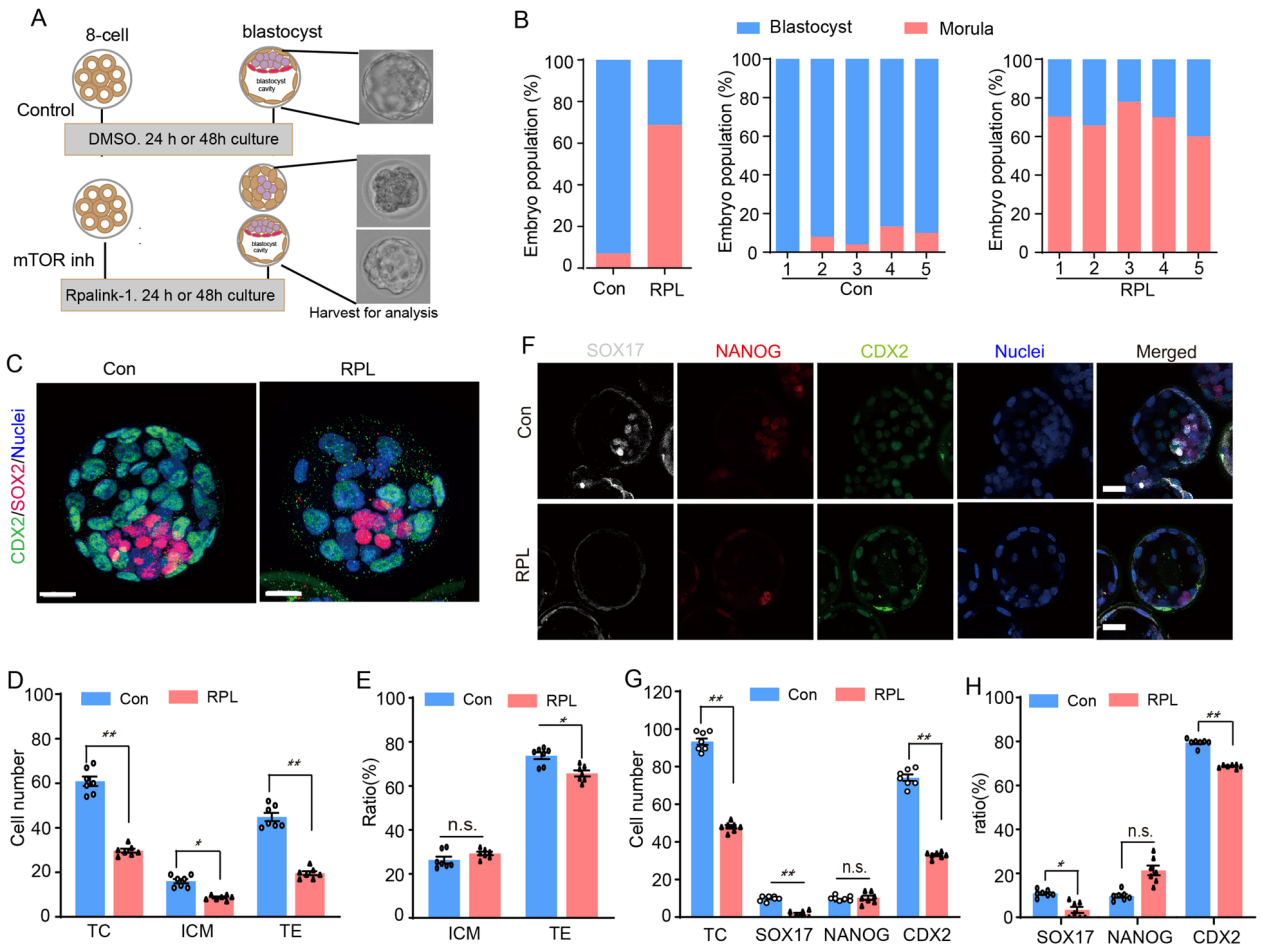
Inhibition of mTOR signaling by Rapalink-1 at the 8-cell stage impaired the morula to blastocyst transformation and decreased the differentiation of trophectoderm lineage. **A** Schematic of experimental setup whereby 8-cell embryos were treated in mTOR inhibitor. **B** The percentage of morula and blastocyst after 24 h cultured in the different treatment conditions. **C** Representative immunofluorescence images of CDX2 and SOX2 in blastocyst from the RPL treatment and Con group. Scale bars, 20 μm. **D** A significantly decreased the total cell number in mTOR inhibitor treated embryos (n = 7). **E** Rapalink-1 treatment decreased the ratio of TE cells. **F** Representative immunofluorescence images of SOX17, NANOG and CDX2 expression in blastocyst derived from RPL and Con group (n = 7). **G** The cell number of total, SOX17^+^, NANOG^+^ and CDX2^+^ of blastocysts in the RPL and Con group (n = 7). **H** Percentage of SOX17^+^, NANOG^+^ and CDX2^+^ cells of the blastocyst in Con and RPLgroup (n = 7). RPL: Rapalink-1 and Con: Control. Error bars are mean ± SEM. ^*^p < 0.05, ^**^ *p* < 0.01, n.s., not significant (*p* > 0.05)

Since nearly half of the embryos were arrested to the compacted 8-cell and morula stage when mTOR was suppressed at the preimplantation development (Fig. 2B and Additional file 1: Figure S2B), we wondered whether inhibition of mTOR signaling influenced the 8-cell/morula to blastocyst transition. To test this, we treated 8-cell embryos with 200 nM Raplink-1 in KOSM medium for 24 h or 48 h and then assessed the population of blastocyst formation and blastocyst quality (Fig. 4A). As expected, 70% of the embryos arrested in the morula stage after 24 h culture, the percentage of blastocyst in Rapalink-1 treated was significantly lower than control group (92% vs 30%) (Fig. 4B). We also observed a significant decrease in the proportion of blastocysts in the groups treated with rapamycin (RPY, mTORC1 inhibitor) and JR-AB2-011 (JR, mTORC2 inhibitor) compared to the control group. (Additional file 1: Figure S3A, B). We further analyzed the quality of developed blastocysts and found that the inhibition of mTOR signaling did not affect the polarization of ICM and TE in the blastocyst, which showed a normal distribution pattern of SOX2 and CDX2 (Fig. 4C). However, the total cell number, ICM cell number, TE cell number and the ratios of TE in blastocyst developed with Rapalink-1 treated decreased significantly when compared with that of untreated condition (Fig. 4D and E). For RPY or JR treatment, the total number of cells in blastocysts developed with RPY or JR treatment significantly decreased compared to the control group. However, there were no significant differences in the number of ICM cells, TE cells, and the ratios of TE (Additional file 1: Figure S4A, B).

By assessing the expression patterns of NANOG/ CDX2/ SOX17, we also observed that inhibition of mTOR signaling can impair CDX2 and SOX17 expression (Fig. 4F), the SOX17^+^ and CDX2^+^ cells number (Fig. 4G) and ratios (Fig. 4H) were significantly lower than those of without mTOR inhibitor treated embryos. However, the inhibition of mTOR signaling did not altering NANOG expression. In summary, these results demonstrate that suppression of mTOR signaling by Rapalink-1 disrupts the balance of ICM/TE commitment and impairs trophoblast differentiation, which underscore the crucial role of mTOR signaling in determining cellular fate.

### mTOR suppression causes changes in gene expression related to trophectoderm cell differentiation and lysosome pathway

**Fig. 5:**
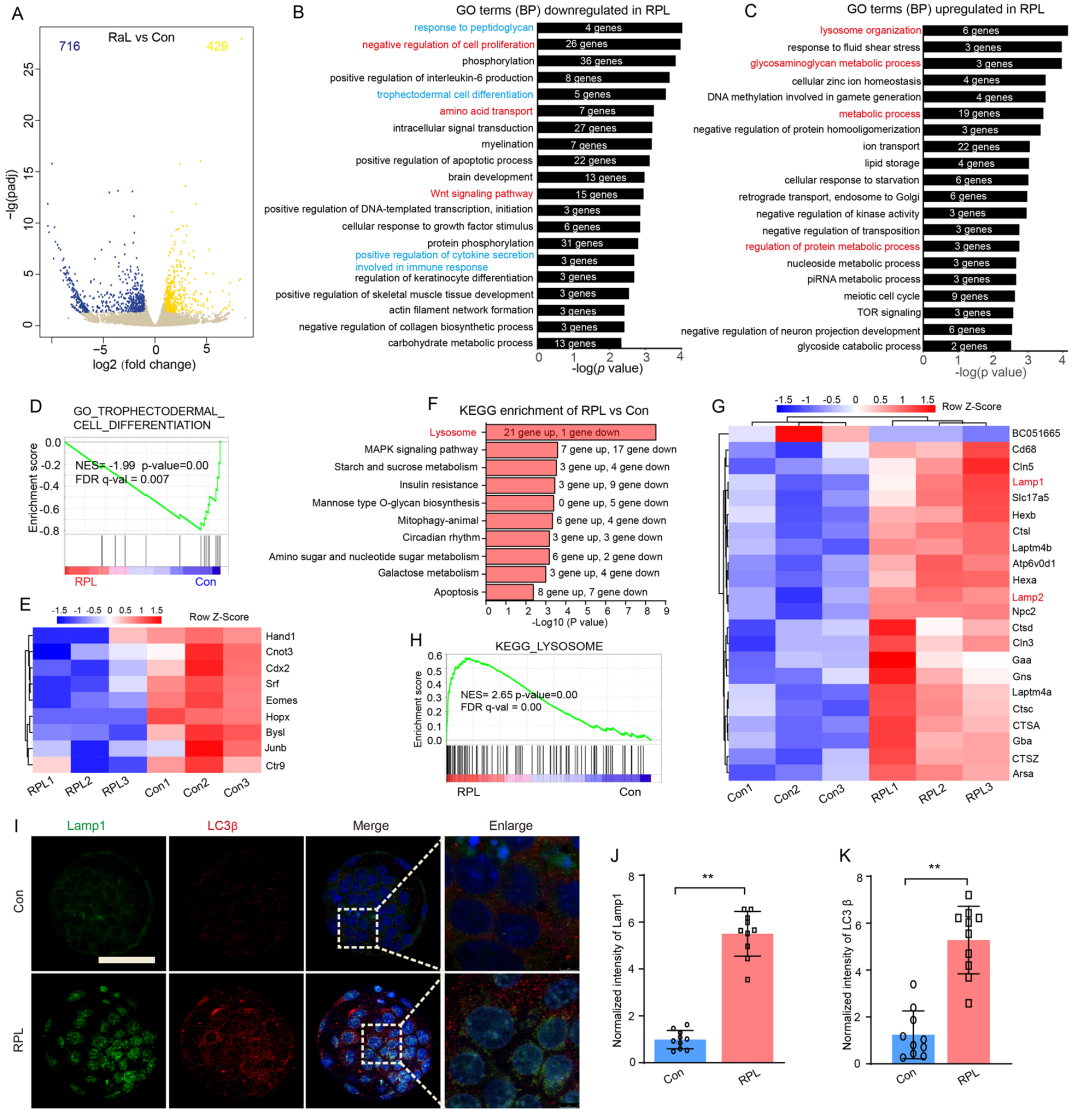
Transcription profiles of blastocysts and key gene terms enriched in mTOR inhibitor treatment during 8-cell to blastocyst development. **A** Volcano plot displaying the DEGs in RPL blastocysts. **B** Bar chart illustrating the enriched GO terms associated with the significantly downregulated transcripts in RPL blastocysts identified by RNA-Seq. **C** Bar chart illustrating the enriched GO terms associated with the significantly upregulated transcripts in RPL blastocysts. **D** GSEA plots showing trophectodermal cell differentiation enriched in RPL-blastocyst (left) and Con-blastocyst (right). NES, normalized enrichment score; FDR, false discovery rate. **E** Heatmap illustrating the relative expression level (row-scaling) of the genes associated with trophectodermal cell differentiation between RPL and Control blastocyst. **F** Bar chart illustrating the top ten enriched KEGG terms or canonical pathways in RPL blastocyst. **G** GSEA profiles showing the most significant enrichment of gene sets associated with lysosomes in RPL blastocysts (RPL vs Con). **H** The heatmap shows the expression pattern of lysosome-related genes in RPL and Con blastocysts. **I** Representative immunofluorescence images of lamp1 (lysosomal associated membrane protein 1) and LC3β (microtubule-associated protein 1 light chain 3β) in RPL and Con embryos. Bar, 50 μm. **J** The normalized intensity of lamp1 between Con and RPL group embryos (n = 10). **K** The normalized intensity of LC3β between Con and RPL group embryos (n = 10)

To understand the molecular alternations of mTOR inhibition on preimplantation embryonic development, SMART2 single cell RNA-seq was used to determine the transcriptomes of blastocysts developed from Raplink-1 (RPL) treated and untreated (Con) embryo (Additional file 1: Figure S5A). In blastocyst, a total of 1145 differentially expressed genes (DEGs) were identified in RPL vs Con group embryos (429 DEGs up-regulated and 716 DEGs down-regulated) (Fig. 5A and Additional file 1: Figure S5B). GO enrichment analysis showed that the DEGs down-regulated were enriched not only the known biological pathways in cells for mTOR suppression, including regulation of cell proliferation, amino acid transport, intracellular signal transduction and Wnt signaling pathway [28, 29], but also special pathways, such as response to peptidoglycan, positive regulation of interleukin-6 production and trophectodermal cell differentiation (Fig. 5B), while up-regulated DEGs were mainly enriched in lysosome organization, metabolic process and ion transport (Fig. 5C and Additional file 1: Figure S5C and D). Gene set enrichment analysis (GSEA) of our RNA-seq data also showed a marked downregulation of trophectoderm cell differentiation-associated genes in the RPL treated embryo (Fig. 5D), including Hand1, Cnot3, Cdx2, Srf and Eomes (Fig. 5E), which have been reported to play critical roles for cell fate commitment and trophoblast development [30]. Additionally, KEGG analysis indicated that these DEGs were mainly involved in lysosome, MAPK signaling pathway, starch and sucrose metabolism, insulin resistance and mannose type O-glycan biosynthesis (Fig. 5F and Additional file 1: Figure S5G and H). Remarkably, the lysosome-related genes, such as Lamp1, Lamp2, Ctsd, Atp6v0d1, Ctsz, Ctsa, Npc2, Ctsl, Laptm4b, Arsa and Hexa, are significantly up-regulated in RPL blastocyst (Fig. 5G). Consistently, GSEA also revealed that the lysosome pathway is the most significantly enriched (Fig. 5H).

We use LysoTracker to measure the effect of mTOR inhibitor treatment on lysosomal activity in embryos. The results indicate that RPL and RPY embryos exhibit significantly increased lysosomal fluorescence signals compared to the control group, while the JR group shows no significant changes (Additional file 1: Figure S6A and B). Furthermore, we used immunofluorescence analysis to study the expression of lamp1 (lysosomal associated membrane protein 1), lamp2 (lysosomal associated membrane protein 2) and LC3 (microtubule-associated protein 1 light chain 3) in blastocyst with RPL treatment, which plays a crucial role in lysosome biogenesis and autophagy [31]. The results showed that the intensity of lamp1 and LC3β immunofluorescence significantly increased in RPL treated developed blastocyst (Fig. 5I–K). Additionally, the expression level of lamp2 is also markedly higher in RPL treated embryos than in Con embryos (Additional file 1: Figure S7). This result suggests that the inhibition of mTOR enhances the activation of lysosomes-autophagy in preimplantation embryos. This observation implies that lysosomes serve as a pivotal signaling center for the embryo’s response to the suppression of mTOR.

### Genome-wide DNA methylation analysis profiles and integrative analysis with transcriptomic data

**Fig. 6:**
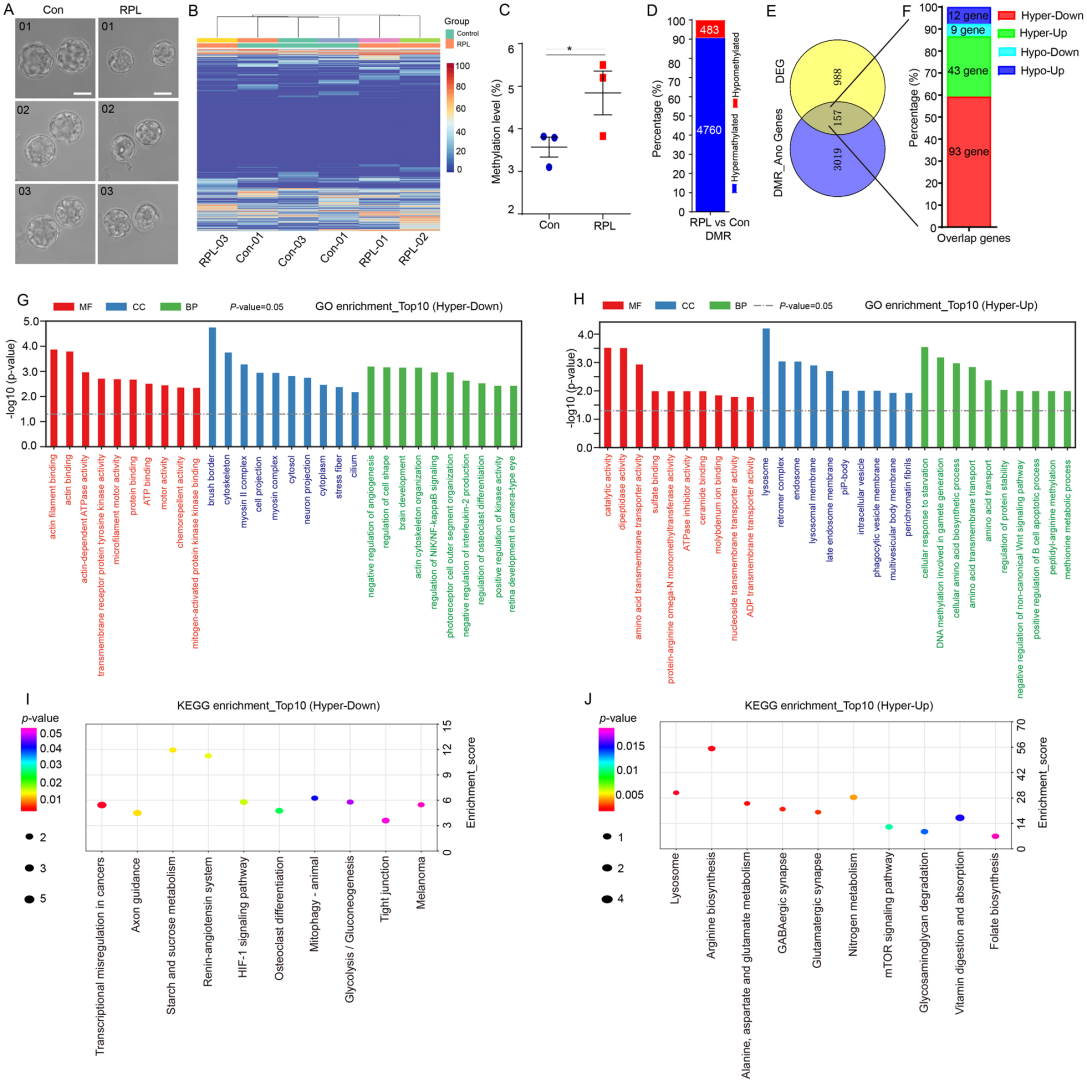
Combined analysis of DNA methylation pattern and transcriptional expression of blastocysts derived from mTOR inhibitor treatment during 8-cell to blastocyst development. **A** The morphology of blastocysts from the control (Con) and rapalink-1 (RPL) groups that were used for genome-wide bisulfite sequencing. **B** Heatmap showing the level of DNA methylation of the CpG site in each group of blastocysts. **C** The levels of genome-wide DNA methylation in RPL and Con are shown (n = 3). **D** The number and proportion of hypermethylated and hypomethylated differentially methylated regions (DMRs) in RPL vs Con. **E** Venn diagram of DMRs-associated genes (DMGs) and differentially expressed genes (DEGs) detected by pair-wise comparison. **F** Bar chart illustrating the number and proportion of hypermethylated-downregulated genes (Hyper-down), hypermethylated-upregulated genes (Hyper-up), hypomethylated-downregulated genes (Hypo-down) and hypomethylated-upregulated genes (Hypo-up) from overlapped genes in DMGs and DEGs. **G** Bar chart illustrating the enriched top GO terms associated with the overlapped genes of hyper-down. **H** Bar chart illustrating the enriched top GO terms associated with the overlapped genes of hyper-up. **I** Bar chart illustrating the top 10 enriched KEGG terms or canonical pathways in overlapped genes of hyper-down. **J** Bar chart illustrating the top 10 enriched KEGG terms or canonical pathways in overlapped genes of hyper-up

To further understand the effect of inhibition of mTOR pathway on epigenetic modification, we performed genome-wide DNA methylation profiling using bisulfite sequencing (BS-seq) in blastocyst samples from the Rapalink-1 treated (RPL, n = 3) and control (Con, n = 3) (Fig. 6A). The hierarchical cluster and heatmap revealed differential methylation level of CpG sites in each group embryos (Fig. 6B). The average methylation level of whole genome was significantly higher in RPL blastocysts (4.84 ± 0.8) than that in Con blastocysts (3.57 ± 0.4) (Fig. 6C). We analyzed differentially methylated regions (DMRs) between the RPL and the Con group and 5250 DMRs were identified, of which 4767 (90.8%) were hypermethylated (Fig. 6D, Additional file 2: Table S1). Additionally, 3177 genes related to these DMRs were annotated (Additional file 3: Table S2). Next, the DMR-associated genes (DMGs) were performed GO and KEGG enrichment analysis. Significantly, the pathways associated with hypermethylated genes were found to be related to cytoskeleton, microtubule-based movement, cell junction, gap junction, and adherens junction (Additional file 1: Figure S8). These results raised the possibility that mTOR inhibition may contribute to genomic DNA hypermethylation, which in turn regulates transcriptional repression in embryos.

To better understand the relationship between DNA methylation and gene expression in mTOR inhibitor treated embryos, we performed the integrated analysis of methylomic and transcriptomic data. The Venn diagram analysis of the DMGs and DEGs showed that 157 overlapping genes were identified in the RPL vs Con group (Fig. 6E). Among the 157 overlapped genes, 93 genes are hyper-down (hypermethylated DMGs and downregulated DEGs), 43 genes are hyper-up (hypermethylated DMGs and upregulated DEGs), 9 genes are hypo-down (hypomethylated DMGs and downregulated DEGs) and 12 genes are hypo-up (hypomethylated DMGs and upregulated DEGs) (Fig. 6F, Additional file 4: Table S3). GO analysis of the overlapped genes with hyper-down showed that the regulation of cell shape, actin cytoskeleton organization and positive regulation of kinase activity were enriched (Fig. 6G), while genes with hyper-up were enriched in the cellular response to starvation, DNA methylation involved in gamete generation, cellular amino acid biosynthetic process and amino acid transport (Fig. 6H). Moreover, KEGG pathway analysis revealed that the terms of starch and sucrose metabolism, HIF-1 signaling pathway, and mitophagy were significantly enriched in genes with hyper-down (Fig. 6I), the enriched pathway for genes of hyper-up were involved in lysosome, arginine biosynthesis, mTOR signaling pathway and vitamin digestion and absorption (Fig. 6J).

### mTOR inhibition induce cytoskeleton disorganization and apoptosis in preimplantation embryos

**Fig. 7:**
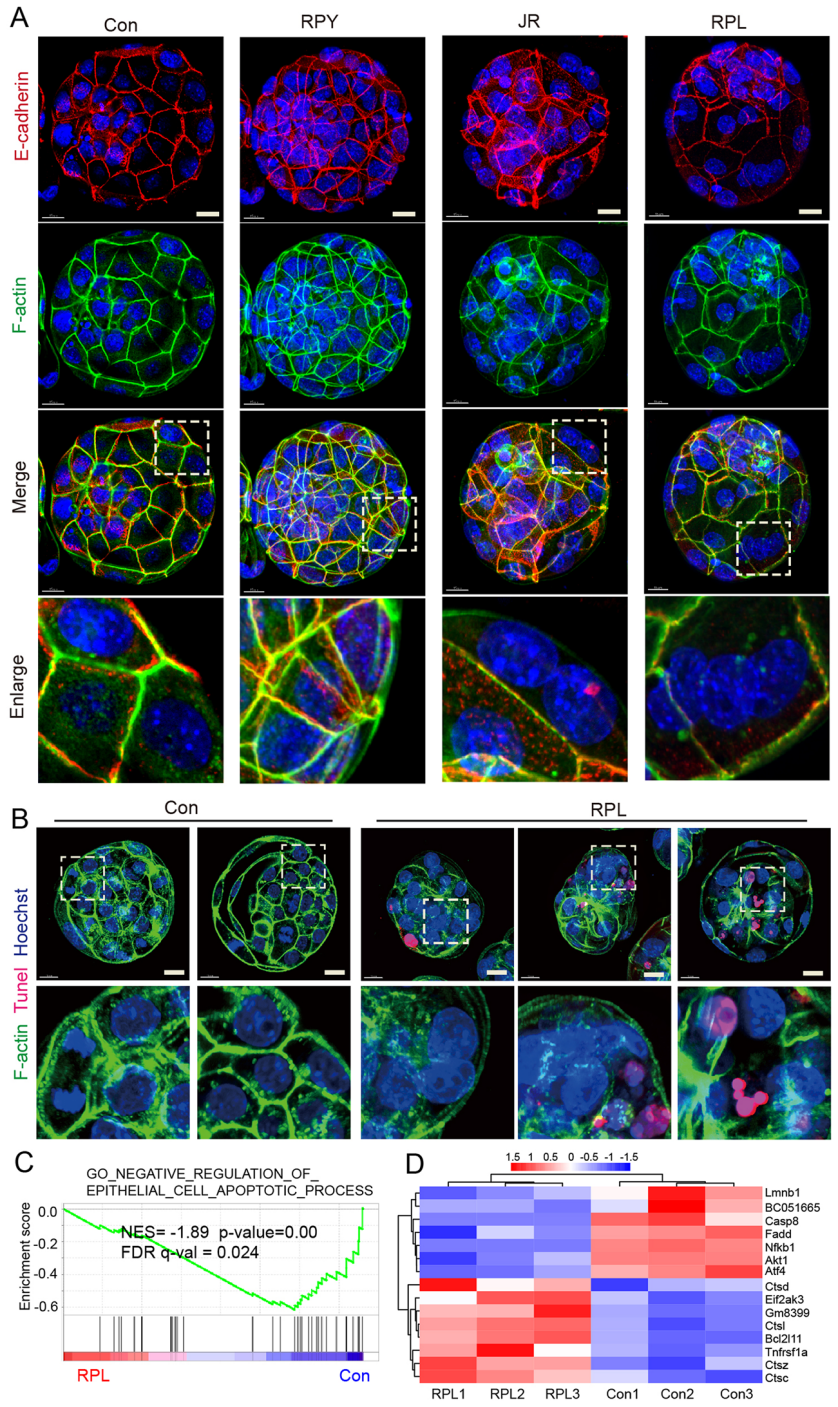
mTOR inhibition in preimplantation embryo disrupt cytoskeletal organization, and lead to blastomeres apoptosis. **A** FITC-phalloidin and E-cadherin immunofluorescence analysis to detect F-actin (green) and E-cadherin (red) in control (Con), rapamycin (RPY), JR-AB2-011 (JR) and Raplink-1 (RPL) treated embryos at 8-cell stages for 24 h development. Bar, 15 μm. **B** Immunofluorescence analysis to detect F-actin (green) and TUNEL staining to detect apoptosis in the blastocysts of control and/or rapalink1-treated groups. Nuclei (blue) are stained with DAPI. Bar, 15 μm. **C** GSEA profiles showing a significant enrichment of gene ontology associate with negative regulation of epithelial cell apoptotic process in RPL and control blastocysts (RPL vs Con). **D** The heatmap shows the expression pattern of apoptosis-related genes in RPL and Con embryos

Our results of GO analysis showed that some DEGs and DMGs were functionally associated with the “actin filament binding”, “actin filament network formation” and “cell junction” clusters (Additional file 1: Figure S5 E and F, Additional file 5: Table S4). Given that mTORC2 control actin cytoskeleton [32] and actin polymerization in mammalian cells [33], we wondered whether mTOR inhibition might influence on cytoskeleton organization in embryos during 8-cell to blastocyst stage and disrupt the actin polymerization. Thus, we used FITC-phalloidin and E-cadherin immunofluorescence analysis to detect the distribution of F-actin, filamentous actin and junction adherents in RPY, JR, RPL treated blastocyst and control embryo. As expected, we found that JR and RPL treated embryos displayed aberrant expression and localization of the cytoskeleton (Additional file 1: Figure S9) and some blastomeres showing accumulated in a separate cytoskeleton network, and the cell-contact was destroyed or cytokinesis failure in those RPL embryos (Fig. 7A), but not in rapamycin and control group embryos. It was also observed that the density of actin filaments was significantly reduced in RPL embryos compared to that of control embryo, and the integrity of the cytoskeleton exhibited deformed, especially actin organization, was disrupted in those apoptotic blastomeres (Fig. 7B, Additional file 1: Figure S10).

Additionally, the GSEA plot also showed that a marked negative regulation of epithelial cell apoptotic process associate genes in RPL embryos (Fig. 7C). The detailed GSEA analysis of each embryo is presented as a heatmap (Fig. 7D), including lysosome activation and expression of cathepsin family genes (Ctsc, Ctsd, Ctsl, and Ctsz) showed remarkably increases in RPL embryos. Our results suggest that the mTOR inhibition as a result of cytoskeleton disorganization, cytokinesis failure in the outside blastomeres and cell apoptosis.

## Discussion

In the current study, we demonstrate that inhibition of mTOR signaling during preimplantation decrease the rate of blastocyst and the competency of implantation, impairs the post implantation embryonic development. Furthermore, we found that inhibition of mTOR signaling compromised the 8-cell/morula to blastocyst transformation and the blastocyst quality, including impairment of trophoblast differentiation and disruption of the balance of ICM/TE commitment. The transcriptome landscapes showed distinct characteristics in blastocysts treated with mTOR inhibitors, such as mTOR suppression marked downregulation of trophectoderm cell differentiation-associated genes in embryo. Methylomic data revealed that inhibiting mTOR led to hypermethylation of genomic DNA in blastocysts. Importantly, we found that mTOR inhibition contributes to increased autophagic-lysosomal activity and disrupted cytoskeleton disorganization and cytokinesis failure in developing embryos.

It was reported that the components of mTOR pathway are expressed and phosphorylated in all stages of preimplantation embryos [34], which are consistent with our immunofluorescence data. Our studies extended these observations, showing mTOR inhibitor treatment for pregnancy mouse cause a significant decreased both the level of P-4E-BP1 and P-AKT of the embryos. Of note, previous work has suggested that homozygous mTOR mutation cause embryonic lethality shortly after implantation due to impaired cell proliferation in both embryonic and extraembryonic compartments [21, 22]. Our results demonstrate that mTOR inhibition significantly decreased proliferative ability of the ICM, the TE and the total cell number of blastocyst, thereby impairing the embryonic development in postimplantation.

Recent an interest study addressed a novel role of glucose metabolism in driving embryogenesis and cell fate specification. In the absence of glucose, embryos become arrested at the compacted morula stage and fail to form a blastocyst due to deposited the synthesis of metabolites derived from hexosamine biosynthetic pathway (HBP) and pentose phosphate pathway (PPP) [5]. In response to HBP, PPP or glucose removal, Cdx2 expression in the TE is reduced, while Oct4 and Nanog levels remained unchanged. In line with the previous study, we demonstrated that suppression of mTOR signaling mainly impaired the 8-cell/morula to blastocyst transformation and expression of the TE-specific transcription factors (Hand1, Cnot3, Cdx2, Srf and Eomes) resulting in defective trophoblast development. However, we found that mTOR inhibition did not affect the development of ICM, and the expression of pluripotent genes (Oct4 and Sox2) were not significant change in those mTOR inhibitor treatment developed blastocysts. Recent emphasis on the importance of TE quality for implantation rates emphasizes the need to make more information available about critical factors for TE differentiation [35]. Besides the first differentiation event, we have also checked the status of differentiation of the primitive endoderm (PrE) and epiblast (EPI) lineages of the blastocyst. Although NANOG expressing cells emerge in the correct proportions, there are very few cells that express SOX17. This suggests that abnormal SOX17 expression may impair the induction of PrE and initiate PrE maturation [36–38]. As an extraembryonic cell lineage, PrE plays an essential role in body axis specification and growth by providing a nutrient supply until placenta formation [39]. Our results indicate that inhibiting mTOR signaling primarily affects the determination of trophectoderm cell fate in embryos.

The connection between mTOR signaling inhibition and altered cell fate determination can be explained from various perspectives. First, inhibition of mTOR signaling disrupted the cellular energy metabolism and intracellular homeostasis, such as indicated by decreased amino acid transport [34, 40], downregulated response to glucose metabolic process or growth factors stimulus [5, 41, 42], impaired cytoskeleton rearrangement [32] and increased lysosome biogenesis [43]. Interestingly, we identified differential activity of lysosomes-autophagy in preimplantation embryos between mTOR treatment and without treatment, demonstrating that proper expression of both LAMP1 and LAMP2 is very important for preimplantation embryonic development [44]. Consistently, it was recently reported that during starvation, mTOR is repressed through LKB1-AMPK that leads to a reduction of metabolically levels and epigenetically silenced in blastocysts [19]. During the normal development of pre-implanted embryos, lysosomal autophagy activity is dynamic. It is high before the 8-cell embryo stage but decreases sharply during the morula and blastocyst stages [45]. Therefore, we believe that excessive lysosomal autophagy during the formation stages from 8 cells to blastocyst is harmful to embryonic development.

Second, inhibition of the PI3-K/Akt/mTOR signal decreased cell proliferation and increased the percentage of apoptotic TE cells [11, 34]. Interestingly, we find that inhibiting the activity of mTORC1 by Rapamycin or mTORC2 by JR-AB2-011 alone did not significant affect cell proliferation and differentiation during preimplantation, suggesting that the distinct roles and the combined effect of mTORC1 and mTORC2 in the embryo development. Third, inhibiting mTOR signaling leads to changes in gene expression that can directly regulate cell differentiation fate. We found that the inhibition of mTOR during embryo development resulted in a decreased expression of various transcripts, including Wnt3a, Axin2, Tfeb, and Tfe3, according to our transcriptomic data. Previous studies have confirmed the involvement of the Wnt signaling pathway in regulating trophectoderm lineage differentiation during embryonic development [46]. Deletion of Tfeb, a transcription factor sensitive to nutrients, has also been shown to induce defects in endodermal differentiation, indicating an intimate connection between Tfeb/lysosomes and cell fate determination [47]. Additionally, a recent study has shown that the Wnt signaling pathway leads to the movement of TFEB into the nucleus, and that TFEB-β-catenin-TCF/LEF is involved in regulating the expression of Wnt target genes [48].

The mTOR regulatory pathway is connected to epigenetic modifications and cytoskeleton rearrangements [19]. Previous studies have demonstrated that inhibiting TOR in Arabidopsis affects plant growth by influencing overall DNA methylation levels and altering DNA methylation patterns of genes involved in plant hormone signal transduction [49]. Our research reveals that mTOR inhibitors cause widespread hypermethylation throughout the blastocyst genome. This hypermethylation leads to the upregulation of specific genes, particularly those relating to actin filament binding, cytoskeleton, and actin cytoskeleton organization. Interestingly, the cytoskeleton has been identified as a second target for the epigenetic machinery of the cell. Several epigenetic' readers, writers, and erasers' that remodel chromatin have been identified [50]. These findings imply a connection between mTOR-mediated cytoskeleton reorganization and epigenetic regulation in preimplantation development.

## Conclusions

In summary, our study demonstrated that mTOR suppression during preimplantation decreases the rate of blastocyst formation and the competency of implantation, impairs the post implantation embryonic development. We found that inhibition of mTOR signaling compromised the 8-cell to blastocyst transformation and the embryo quality, including impairment of trophectoderm cell differentiation. mTOR suppression significantly changes the transcriptome and methylome landscape of embryos. Our findings highlight lysosome-autophagy activation and cytoskeleton remodeling as a molecular link in explaining how adaptations of embryos response to mTOR suppression, which may compromise embryo quality.

### Supplementary Information

**Additional file 1: Figure S1.** The dynamics expression pattern of mTOR, pS6 and p-AKT at different stage of preimplantation development in mice. **Figure S2.** Dose-dependent effects of short-term exposure of mTOR inhibitors for blastocyst formation. **Figure S3.** Inhibition of mTOR signaling by Rapamycin, JR-AB2-011 and Rapalink-1 at the 8-cell stage impaired the morula to blastocyst transformation. **Figure S4.** Effect of mTOR suppression by Rapamycin, JR-AB2-011 and Rapalink-1 at the 8-cell stage on the differentiation of trophectoderm lineage. **Figure S5.** Blastocysts from Con and the RPL treated group were subjected to smart2-seq single-cell transcriptome analysis. **Figure S6.** Lysosome profiles in mTOR inhibitors treatment during 8-cell to blastocyst development. **Figure S7. **Representative immunofluorescence images of lamp2 (lysosomal associated membrane protein 2) and F-actin in RPL and Con embryos. **Figure S8.** GO and KEGG enrichment analysis of differentially methylated regions (DMRs)-associated genes in blastocysts from Con and the RPL treated group.** Figure S9.** Effect of mTOR inhibition in preimplantation embryo on cytoskeletal organization. **Figure S10.** Aberrant expression and localization of the cytoskeleton in JR-AB2-011 (JR) and Raplink-1 (RPL) treated embryos.

**Additional file 2:** **Table S1.** Differentially methylated regions (DMRs) between the RPL and the Con group.

**Additional file 3: Table S2.** Genes annotated that related to DMRs.

**Additional file 4: Table S3.** The Venn diagram analysis of the DMGs and DEGs for methylomic and transcriptomic data.

**Additional file 5: Table S4.** GO analysis of DEGs and DMGs for methylomic and transcriptomic data.

## Data Availability

All data presented in the current study are available from the corresponding author on reasonable request.

## Abbreviations

mTOR: Mechanistic target of rapamycin
ZGA: Zygotic genome activation
mTORC1: Rapamycin-sensitive mTOR complex 1
mTORC2: Rapamycin-insensitive mTOR complex2
ICM: Inner cell mass
TE: Trophectoderm
PMSG: Pregnant mare serum gonadotrophin
hCG: Human chorionic gonadotrophin
dpc: Days post coitum
PFA: Paraformaldehyde
RPL: Rapalink-1
RPY: Rapamycin
JR: JR-AB2-011
IS: Implantation sites
DEGs: Differentially expressed genes
GO: Gene ontology
KEGG: Kyoto Encyclopedia of Genes and Genomes
P-4E-BP1: Phospho-4E-BP1 Thr37/46
P-AKT: Phospho-Akt Ser473
EPI: Epiblast
PrE: Primitive endoderm
GSEA: Gene set enrichment analysis
lamp1: Lysosomal associated membrane protein 1
lamp2: Lysosomal associated membrane protein 2
LC3: Microtubule-associated protein 1 light chain 3
DMRs: Differentially methylated regions
DMGs: DMR-associated genes
