# Supplementary material for: mTOR hypoactivity leads to trophectoderm cell failure by enhancing lysosomal activation and disrupting the cytoskeleton in preimplantation embryo
Source: Cell Biosci. 2023 Nov 30;13:219. doi: 10.1186/s13578-023-01176-3 (PMC10688112; doi:10.1186/s13578-023-01176-3)
Supplement: Supplementary file 1 — Additional file 1: Figure S1. The dynamics expression pattern of mTOR, pS6 and p-AKT at different stage of preimplantation development in mice. Figure S2. Dose-dependent effects of short-term exposure of mTOR inhibitors for blastocyst formation. Figure S3. Inhibition of mTOR signaling by Rapamycin, JR-AB2-011 and Rapalink-1 at the 8-cell stage impaired the morula to blastocyst transformation. Figure S4. Effect of mTOR suppression by Rapamycin, JR-AB2-011 and Rapalink-1 at the 8-cell stage on the differentiation of trophectoderm lineage. Figure S5. Blastocysts from Con and the RPL treated group were subjected to smart2-seq single-cell transcriptome analysis. Figure S6. Lysosome profiles in mTOR inhibitors treatment during 8-cell to blastocyst development. Figure S7. Representative immunofluorescence images of lamp2 (lysosomal associated membrane protein 2) and F-actin in RPL and Con embryos. Figure S8. GO and KEGG enrichment analysis of differentially methylated regions (DMRs)-associated genes in blastocysts from Con and the RPL treated group. Figure S9. Effect of mTOR inhibition in preimplantation embryo on cytoskeletal organization. Figure S10. Aberrant expression and localization of the cytoskeleton in JR-AB2-011 (JR) and Raplink-1 (RPL) treated embryos. [file 13578_2023_1176_MOESM1_ESM.docx]

**Supplement figure and legends**


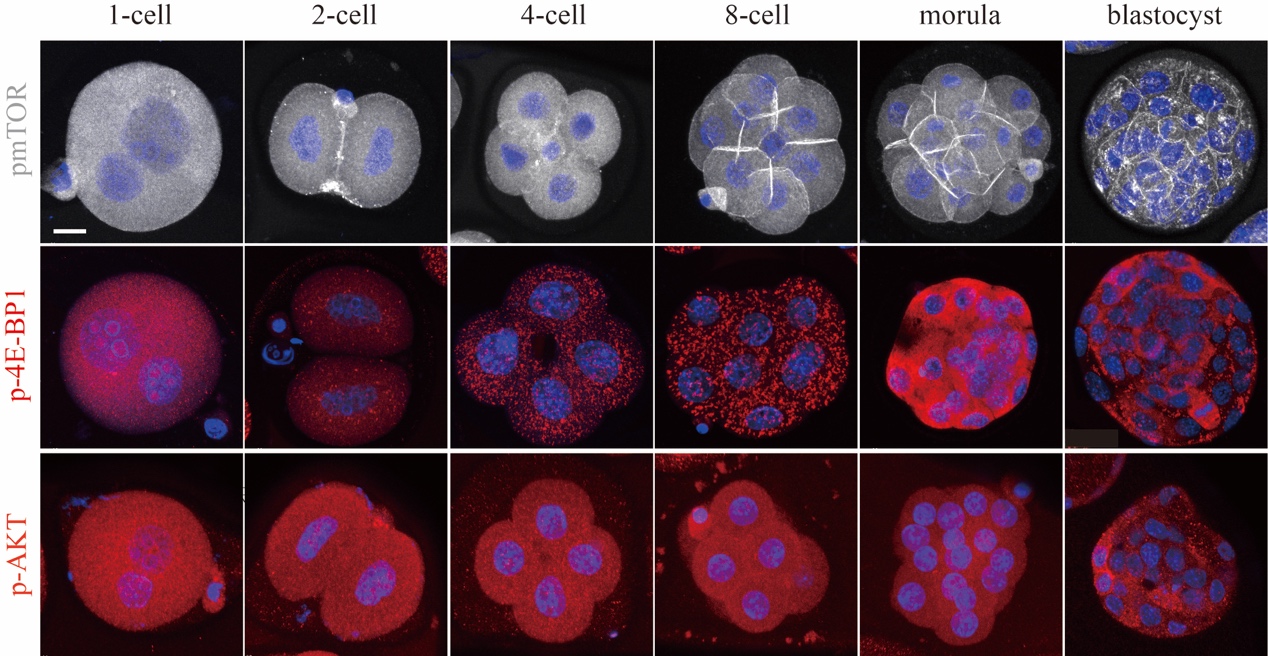


**Figure S1**. The dynamics expression pattern of mTOR, pS6 and p-AKT at different stage of preimplantation development in mice. Localization of mTOR, p-S6 (a target of mTORC1) and p-AKT (a target of mTORC2) in 1-cell, 2-cell, 4-cell, 8-cell, morula and blastocyst was investigated by immunostaining with their antibody and Hoechst nuclear stain respectively. Scale bar: 20 μm.


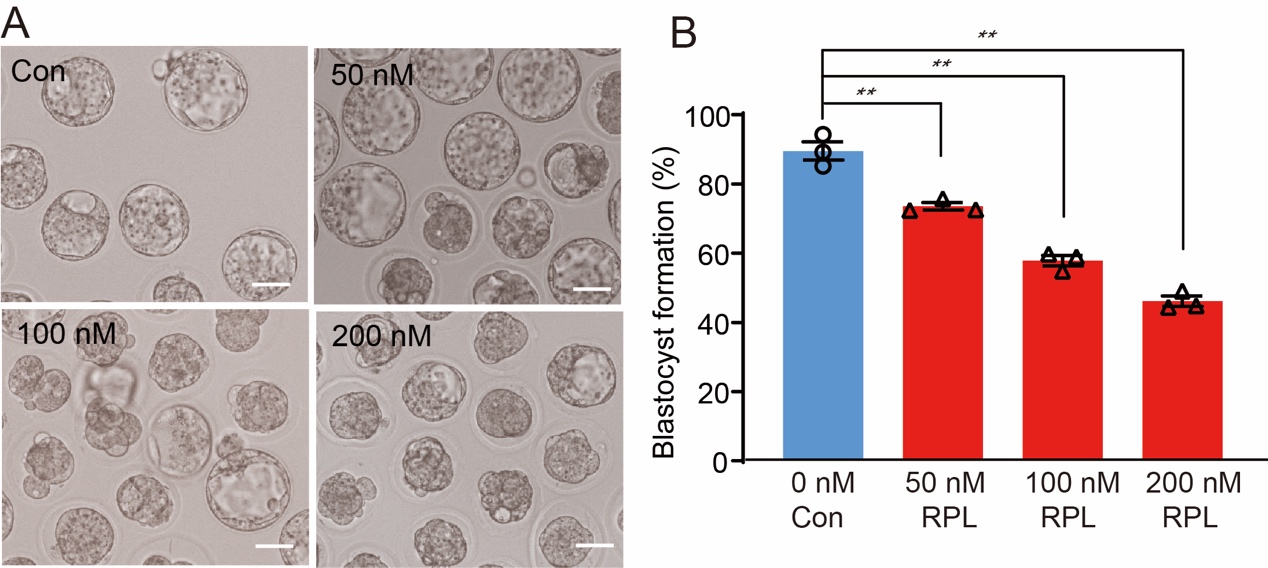


**Figure S2**. Dose-dependent effects of short-term exposure of mTOR inhibitors for blastocyst formation. 1-cell embryos were treated with Rapalink-1 for 24h at different concentrations, and then the embryos were washed and subsequent culture in KSOM medium for 72h without drugs. (A) Representative bright-field images of embryos developed in vitro with 50 nM, 100 nM, 200 nM RPL treated or without treatment. Scale bars, 50 μm. (B) The blastocyst formation rate was calculated. Error bars are mean ± SEM. ** *p*<0.01, * *p*<0.05, n.s., not significant (*p* > 0.05). RPL, Rapalink-1. Con, Control.


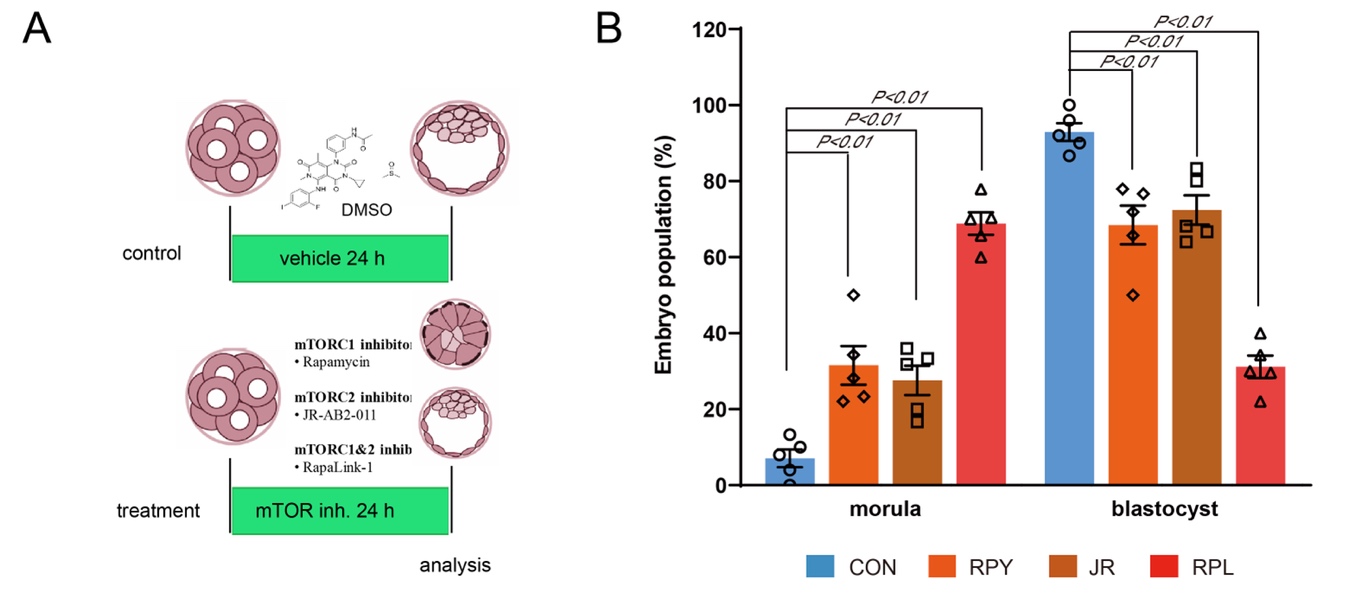


**Figure S3**. Inhibition of mTOR signaling by Rapamycin, JR-AB2-011 and Rapalink-1 at the 8-cell stage impaired the morula to blastocyst transformation. (A) Schematic of experimental setup whereby 8-cell embryos were treated in mTOR inhibitors. (B) The percentage of morula and blastocyst after 24 h cultured in the different treatment conditions. RPY: Rapamycin, JR: JR-AB2-011, RPL: Rapalink-1 and Con: Control. Error bars are mean ± SEM. *p<0.05, ** p<0.01, n.s., not significant (p > 0.05).

**
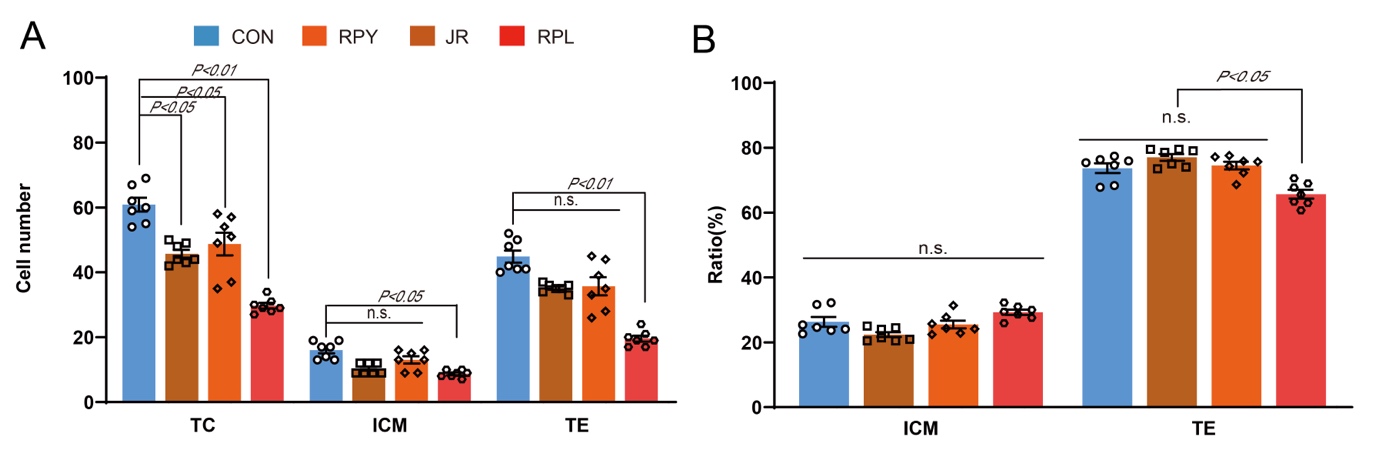
**

**Figure S4**. Effect of mTOR suppression by Rapamycin, JR-AB2-011 and Rapalink-1 at the 8-cell stage on the differentiation of trophectoderm lineage. (A) A significantly decreased the total cell number in mTOR inhibitors treated embryos (n = 7). (B) Rapalink-1 treatment decreased the ratio of TE cells (n = 7). TC: total cell number, ICM: inner cell mass, TE: trophectoderm, RPY: Rapamycin, JR: JR-AB2-011, RPL: Rapalink-1 and Con: Control. Error bars are mean ± SEM. *p<0.05, ** p<0.01, n.s., not significant (p > 0.05).


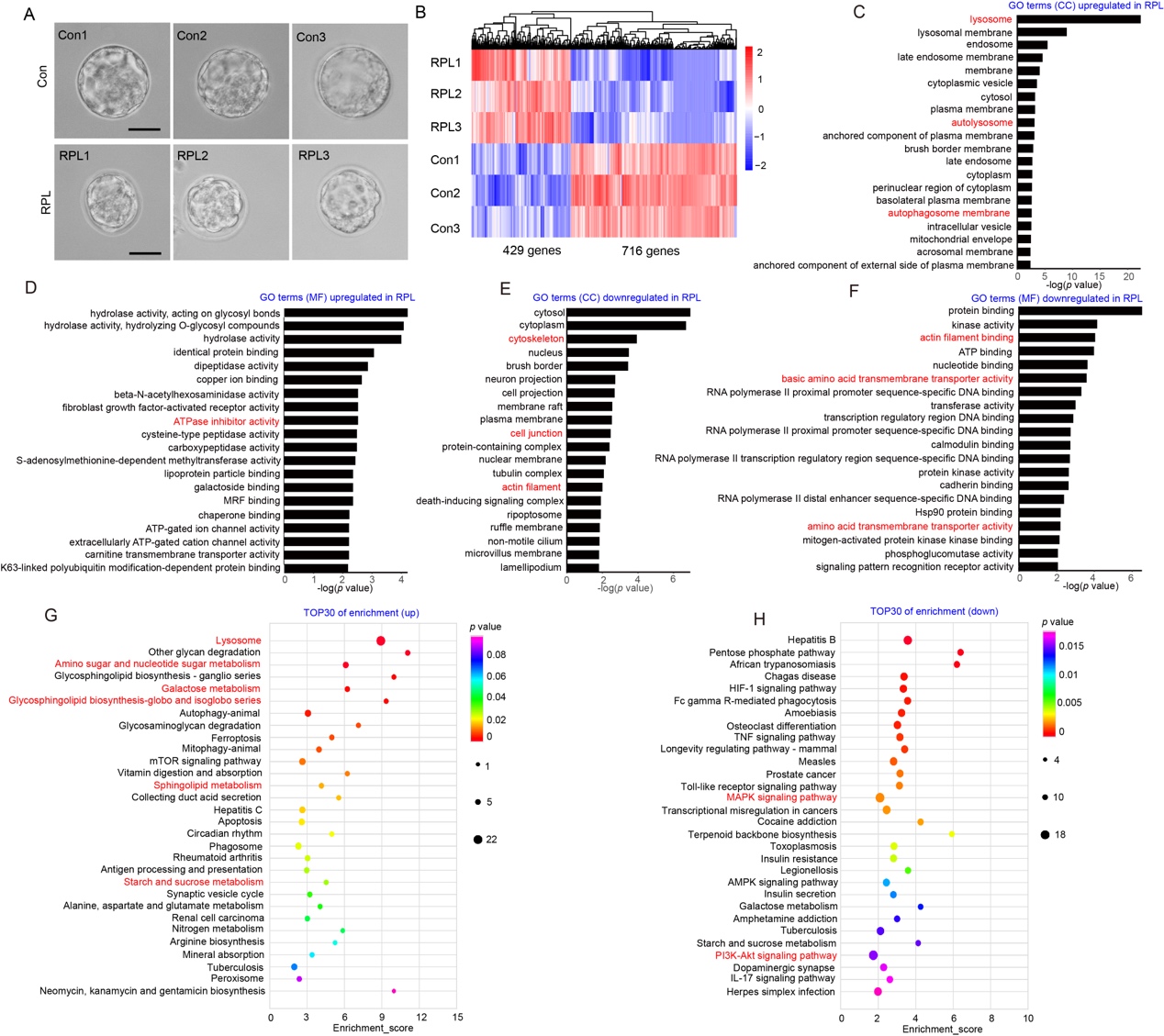


**Figure S5**. Blastocysts from Con and the RPL treated group were subjected to smart2-seq single-cell transcriptome analysis. (A) The morphology of blastocysts from the control (Con), and Rapalink-1 (RPL) groups that were used for single-cell RNA sequencing. Scale bars, 50 μm. Numbers denote the ID of each group of blastocysts. (B) Heatmap showing the different expression genes (DEGs) between RPL blastocysts and Con blastocysts. (C, D) Bar chart shows the top 20 GO terms of up-regulated genes of cellular component (CC) and molecular function (MF) in RPL vs Con. (E, F) Bar chart shows the top 20 GO terms of down-regulated genes of cellular component (CC) and molecular function (MF) in RPL vs Con. (G) Dot plot shows the up-regulated KEGG pathways enriched in RPL vs Con. The size of the dot is based on gene number enriched in the pathway, and the color of the dot shows the pathway enrichment significance. (H) Dot plot shows the down-regulated KEGG pathways enriched in in RPL vs Con.


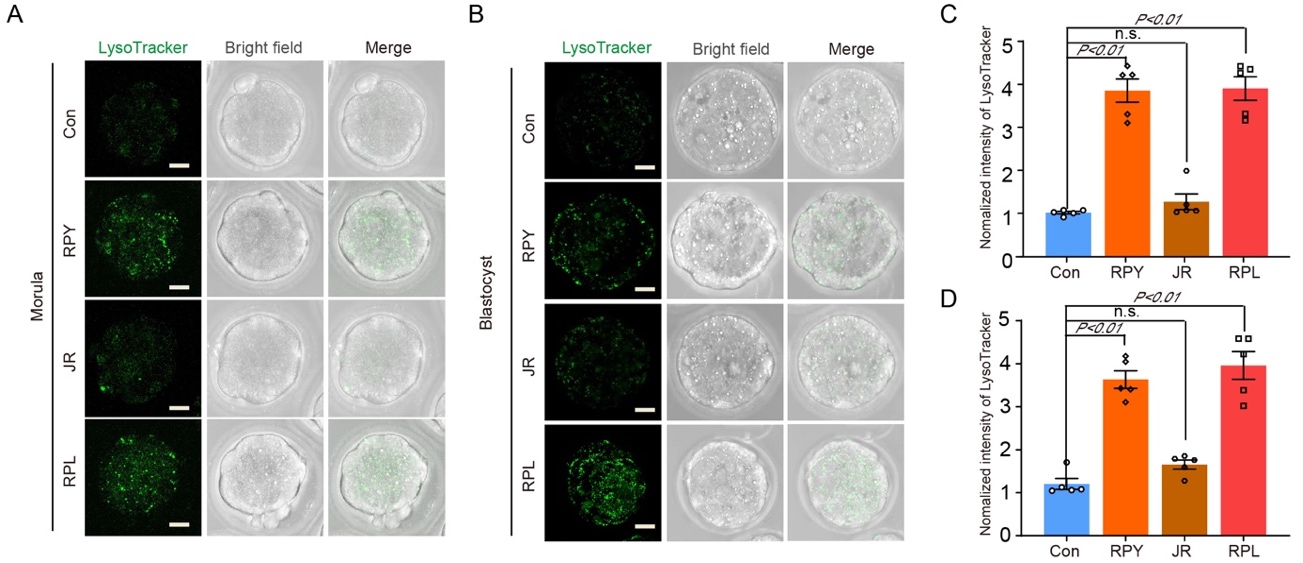


**Figure S6.** Lysosome profiles in mTOR inhibitors treatment during 8-cell to blastocyst development. (A) Representative immunofluorescence images of LysoTracker (labeling and tracing lysosomes in live cell) in RPY, JR, RPL and Con group at morula stage. Bar, 20 μm. (B) Representative immunofluorescence images of LysoTracker in RPY, JR, RPL and Con group at blastocyst stage. Bar, 20 μm. (C) The normalized intensity of LysoTracker in the morula of each group (n = 5). (D) The normalized intensity of LysoTracker in the blastocyst of each group (n = 5). RPY: Rapamycin, JR: JR-AB2-011, RPL: Rapalink-1, Con: Control.


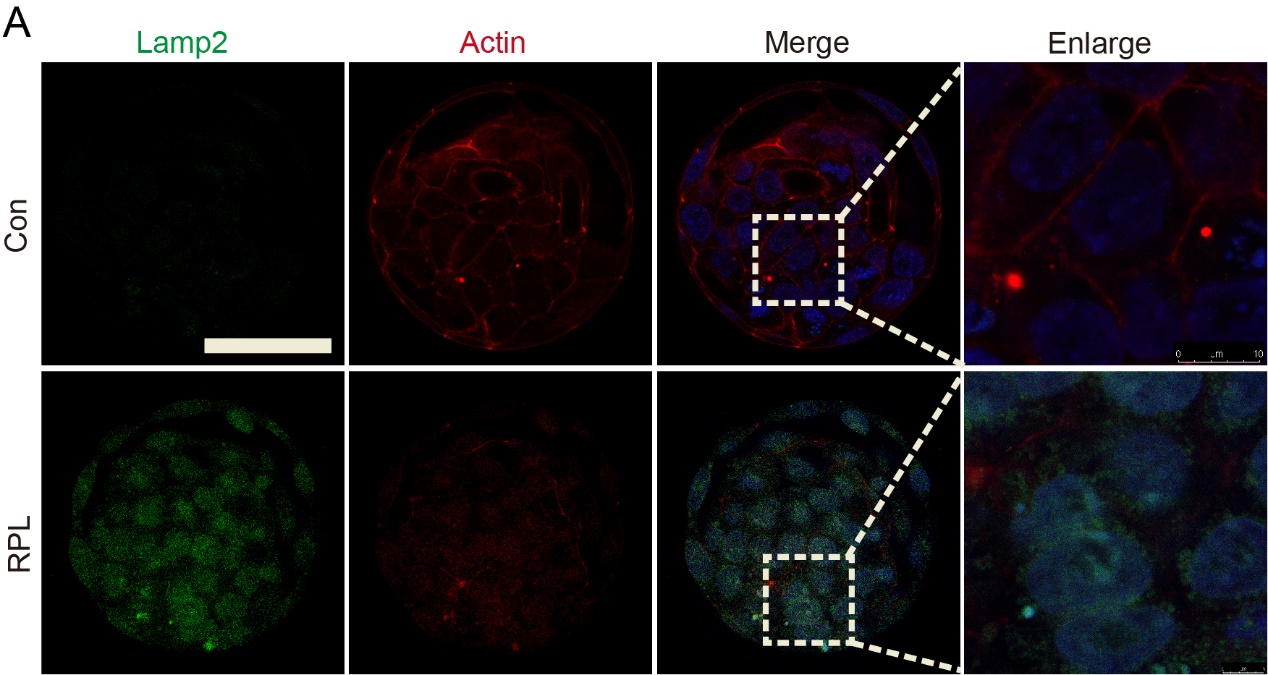


**Figure S7.** Representative immunofluorescence images of lamp2 (lysosomal associated membrane protein 2) and F-actin in RPL and Con embryos. Nuclei (blue) are stained with DAPI. Bar, 50 μm.


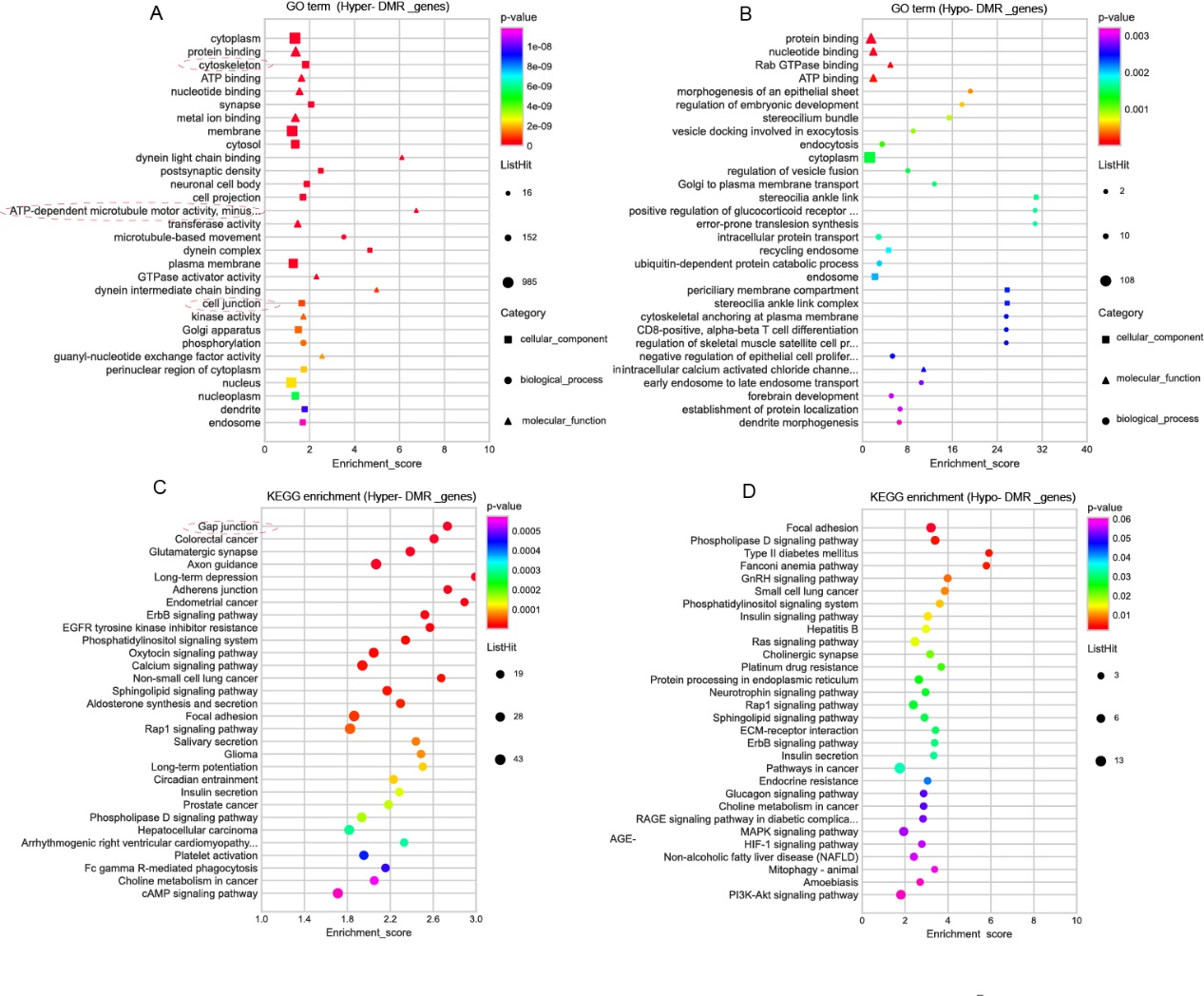


**Figure S8**. GO and KEGG enrichment analysis of differentially methylated regions (DMRs)-associated genes in blastocysts from Con and the RPL treated group.

(A) Dot plot shows the top GO terms of hypermethylated-DMR-associated genes (DMGs) for cellular component (CC), biological process (BP) and molecular function (MF) in RPL vs Con. (B) Dot plot shows the top GO terms of hypomethylated-DMR-associated genes (DMGs) for cellular component (CC), biological process (BP) and molecular function (MF) in RPL vs Con. (C) Dot plot shows the KEGG enriched pathways of hypermethylated DMGs in RPL vs Con. (D) Dot plot shows the KEGG enriched pathways of hypomethylated DMGs in RPL vs Con.


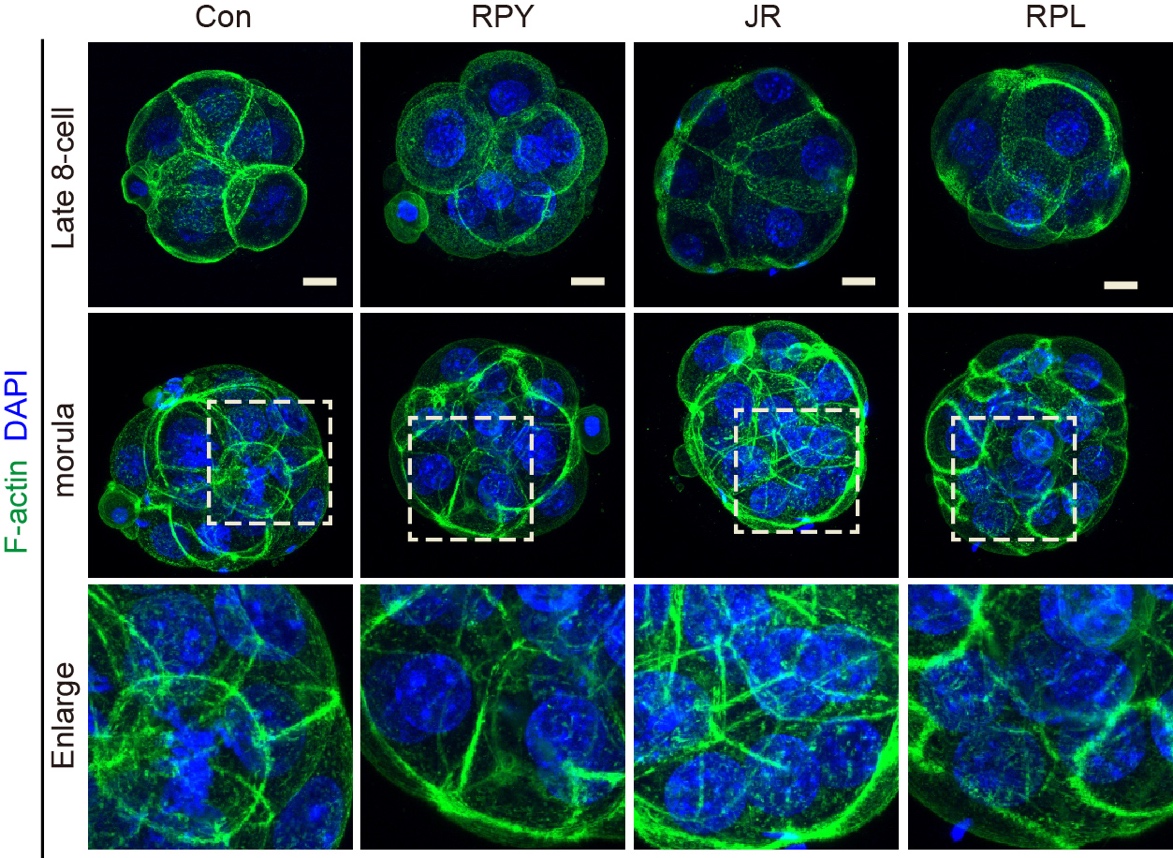


**Figure S9.** Effect of mTOR inhibition in preimplantation embryo on cytoskeletal organization. FITC-phalloidin immunofluorescence staining analysis was performed to detect F-actin (green) in embryos at the early 8-cell stage treated with Con, RPY, JR-AB2-011, and RPL. The treatment duration for sampling was 6 and 18 hours, respectively. Bar, 20 μm.


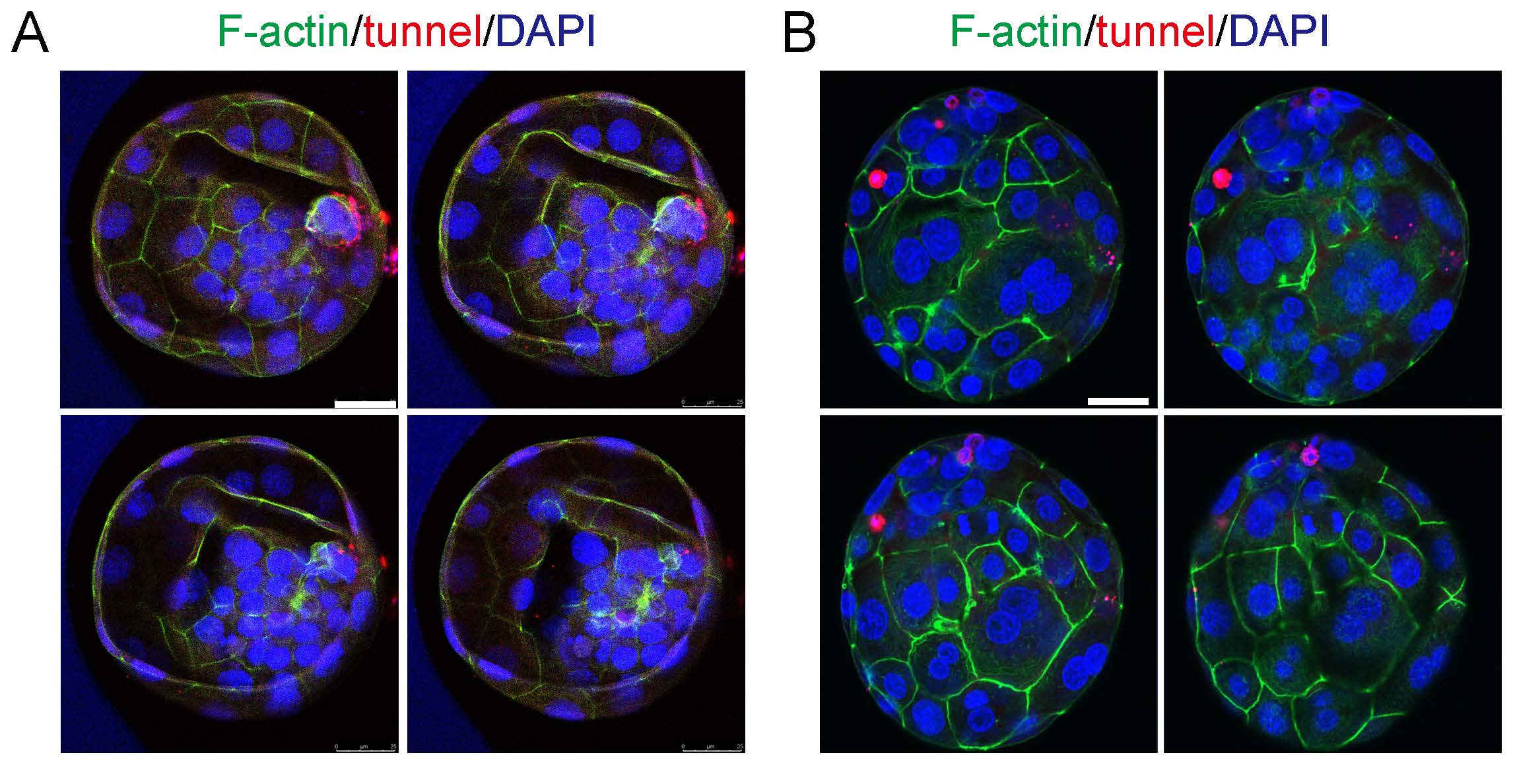


**Figure S10**. Aberrant expression and localization of the cytoskeleton in JR-AB2-011 (JR) and Raplink-1 (RPL) treated embryos. Representative IF staining image of blastocyst derived from JR (A) or RPL treated embryos (B) for F-actin (green) and terminal deoxynucleotidyl transferase-mediated dUTP nick end labeling (TUNEL) (Red). DNA was counterstained with DAPI (blue). Scale bars: 20 μm.
